# Dynamic uncertainty-level assessment framework for real-time needle tracking in CT-guided surgical environments

**DOI:** 10.1007/s11548-026-03725-w

**Published:** 2026-06-05

**Authors:** Max Steiger, Mohammad Rezapourian, Marko Rak, Christian Hansen

**Affiliations:** 1 Otto von Guericke University Magdeburg, Faculty of Computer Science, Chair of Virtual and Augmented Reality, Universitaetsplatz 2, 39106 Magdeburg, Germany; 2 Research Campus STIMULATE, Otto-Hahn-Str. 2, 39106 Magdeburg, Germany

**Keywords:** CT-guided interventions, Needle tracking, Uncertainty quantification, Image-guided surgery, Decision support

## Abstract

**Purpose:**

Accurate needle tracking is critical for the success of computed tomography (CT)-guided interventions, where even minor deviations may compromise procedural safety and clinical outcomes. However, existing image-guided tracking systems typically lack mechanisms to quantify and communicate the reliability of their predictions in real time, leaving clinicians to act on guidance of uncertain trustworthiness.

**Methods:**

We propose a *Dynamic Uncertainty Level Assessment Framework* that provides a quantitative, real-time estimate of tracking reliability by linearly linking a predicted uncertainty score to spatial tracking error. The framework consists of three different approaches: (1) a classic method based on dynamically weighted, interpretable reliability metrics; (2) a lightweight convolutional neural network (CNN) that predicts uncertainty directly from multi-view image data; and (3) a hybrid CNN that adaptively optimizes metric weights while preserving interpretability. The uncertainty level is defined on a fixed scale, with 0% corresponding to ideal tracking (error of 0 mm) and 100% to a tracking error of 10 mm.

**Results:**

Experimental validation on clinical and clinically realistic laboratory datasets of ∼30,000 frames demonstrates a strong positive correlation between uncertainty and error (Pearson r>0.82) and achieves a stable tracking error estimation (error difference <0.6 mm) with real-time performance (5 ms per frame).

**Conclusion:**

By enabling an intuitive uncertainty-to-error mapping, the proposed framework supports more informed intra-procedural decision-making, enhances operator trust in guidance data, and establishes a practical basis for integration into uncertainty-aware CT-guided intervention systems.

**Supplementary Information:**

The online version contains supplementary material available at https://doi.org/10.1007/s11548-026-03725-w.

## Introduction

Computed tomography (CT)-guided interventions, such as percutaneous biopsies and tumor ablations, are widely established procedures in minimally invasive medicine [1]. Their success depends on precise needle placement, requiring accurate real-time tracking to navigate complex anatomy while minimizing procedural impact. In dynamic operating rooms, fluctuating lighting, tissue occlusions, and rapid motion can degrade tracking accuracy [2], potentially leading to misplaced needles, non-diagnostic samples, or unintended tissue injury [3].

Previous needle-tracking approaches have employed traditional image processing, optical and electromagnetic sensors, and deep-learning-based localization [4]. Despite advances, most systems report tracking coordinates without dynamic uncertainty estimates [5, 6], forcing clinicians to rely on outputs even when uncertainties are present. This lack of real-time uncertainty quantification can cause unrecognized inaccuracies and procedural errors.

To address this gap, we present a dynamic uncertainty-level assessment framework for real-time needle tracking in CT-guided interventions. The system outputs the estimated needle position along with a quantitative uncertainty (as a percentage), linearly calibrated to the spatial tracking error: 0% corresponds to ideal tracking (0 mm error), and 100% to a tracking error of 10 mm. This formulation enables surgeons to assess reliability immediately and make informed intraoperative decisions, such as pausing for recalibration or repositioning sensors. We compare three uncertainty-assessment strategies: (1) a classic model using interpretable hand-crafted metrics; (2) an end-to-end CNN predicting uncertainty directly from images; and (3) a hybrid CNN learning dynamic metric weights while preserving interpretability. By linking uncertainty to spatial accuracy through fixed linear calibration, the framework improves transparency, strengthens trust in guidance, and supports safer use in complex surgical workflows.

## Related work

Accurate instrument tracking is essential for CT-guided interventions [7]. While optical, electromagnetic, and deep learning-based solutions have advanced needle localization [7, 8], a persistent limitation is the absence of reliable, real-time confidence quantification [6, 9].

Recent work has explored uncertainty quantification through Bayesian neural networks and deep ensembles, estimating both aleatoric and epistemic uncertainties [10, 11]. Confidence calibration techniques have also been proposed [9]. However, these methods typically output statistical quantities such as predictive variance, entropy, or uncalibrated softmax scores—measures that remain abstract and difficult to interpret clinically. Clinicians cannot readily assess whether a reported uncertainty of, for example, 0.3 variance units corresponds to a tolerable or critical deviation in needle position.

The key gap lies in transforming model-derived uncertainty into a clinically interpretable reliability measure. To address this, our framework computes a real-time uncertainty level $U \in [0,100]\%$ that correlates strongly with physical tracking error. Unlike abstract statistical quantities, this percentage-based representation provides an intuitive, normalized scale (0%: low uncertainty; 100%: critical unreliability) with optional linear mapping to estimated physical error (U/100·10 mm), supporting intraoperative decision-making under occlusions, motion, and illumination changes.

## Materials and methods

To address varying requirements for interpretability and automation, we propose three methods for uncertainty estimation: a classic method using handcrafted metrics for full transparency, a fully CNN-based method that regresses uncertainty directly from images without manual tuning, and a hybrid CNN that learns optimal metric weights while retaining interpretability. Data were acquired using synchronized RGB cameras for needle tracking and ground-truth acquisition (AprilTag for the clinical dataset, Atracsys optical tracker for the semi-clinical dataset), enabling training by correlating tracking outputs, environmental conditions, and actual errors.

### Datasets

The framework was trained and evaluated on two publicly available datasets acquired at the Research Campus STIMULATE, Magdeburg  [12]. The *clinical dataset*, recorded in a CT laboratory replicating real interventional workflows, contains ∼22,000 frames capturing diverse needle manipulation scenarios and realistic user interactions. The *semi-clinical dataset*, acquired under controlled laboratory conditions, includes ∼8000 frames with systematic variations in lighting, occlusion, and motion dynamics. Both datasets utilized five biopsy needles (10–20 cm, 14–18 G) positioned in front of an abdomen phantom with surgical drapes. Ground-truth 3D needle-tip coordinates were acquired using independent reference systems: an Atracsys optical tracker (target registration error (TRE) <0.3 mm) for the semi-clinical dataset and AprilTag-based tracking (TRE <0.7 mm) for the clinical dataset. All frames were processed with the needle-tracking system integrating three software-synchronized RGB cameras (1920 × 1080 px) and a UNetConvNeXt segmentation model [13] for marker-less tip and base estimation (TRE <0.7 mm). Each dataset entry contains synchronized RGB images, predicted 3D needle-tip coordinates, and corresponding ground-truth coordinates. The ground-truth tracking error, computed as Euclidean distance between predicted and reference positions, serves as the training target for the CNN-based methods.

**Fig. 1:**
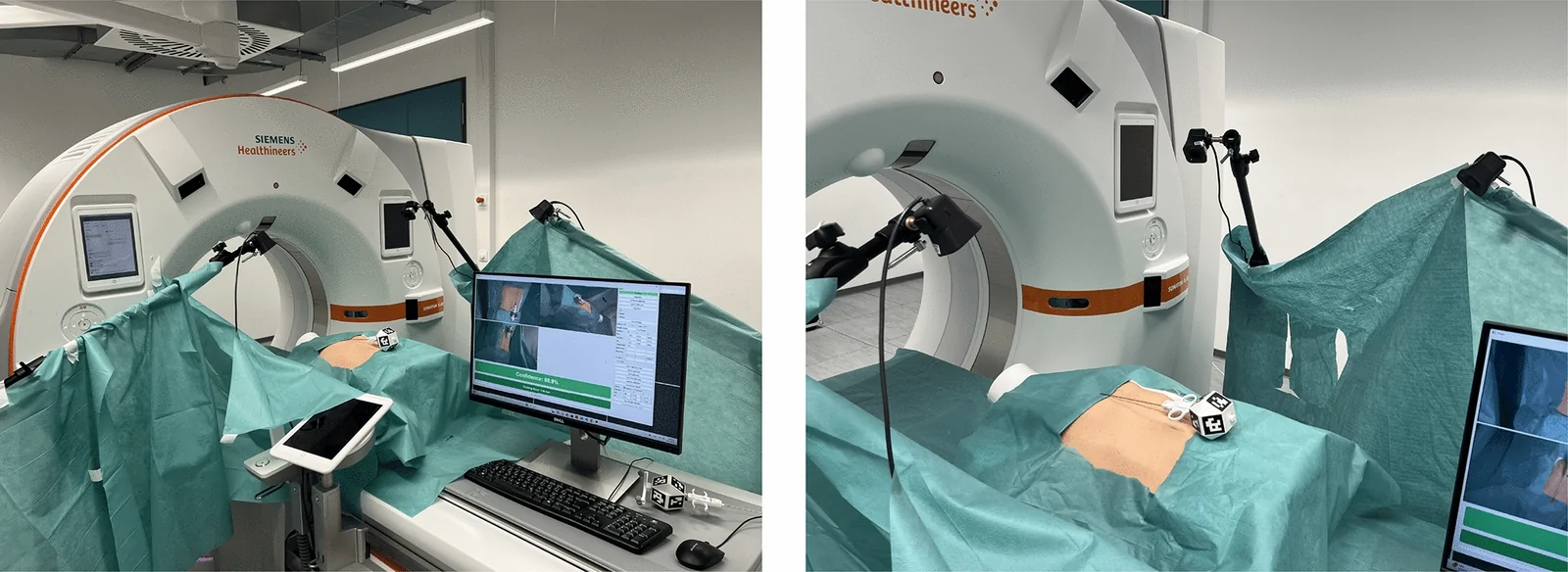
Experimental configuration used for capturing the dataset and evaluating system performance

Paired photometric augmentation (brightness, contrast, gamma jitter, Gaussian noise) was applied to improve robustness. Both datasets were partitioned into training (70%), validation (15%), and test (15%) subsets at the sequence level to prevent data leakage. The full CNN was trained on the combined training partitions ( 21,000 frames), with the validation partitions kept independent for early stopping monitoring. The hybrid CNN, owing to its lightweight architecture ( 0.5M parameters), benefits more from additional training data than from a large independent validation set; therefore, training and validation partitions were pooled ( 25,000 frames), with an internal 90/10 split reserved for early stopping. The classic method requires no training data. All three methods were evaluated on identical held-out test partitions. The experimental setup is shown in Fig. 1.

### System architecture

The needle-tracking system employs three RGB cameras (1920×1080 px) positioned at distances of 50–100 cm from the needle, mounted around the operating table in both datasets. Two cameras are placed on the same side and a third on the opposite side, providing approximately 180$^\circ$ of angular coverage to ensure robust multi-view triangulation (Fig. 1). The cameras are software-synchronized and calibrated using a Charuco board [14], from which both intrinsic parameters and extrinsic parameters (relative camera poses) are obtained.

Each frame is processed with a U-NetConvNeXt segmentation model to localize the needle tip and base via multi-view triangulation. The system outputs the 3D needle position and an uncertainty level $U \in [0,100]\%$, computed using one of three methods (Uncertainty computation methods Section). Regardless of the method, *U* follows the fixed linear calibration (Uncertainty-to-error calibration Section), enabling conversion to an estimated tracking error:

$$\begin{aligned} {\hat{E}} = \frac{U}{100} \cdot 10\,\text {mm}. \end{aligned}$$

These values are displayed in a GUI with a dynamic color bar. The software is implemented in Python, leveraging OpenCV for image preprocessing and PyTorch for GPU-accelerated inference via CUDA.

**Fig. 2:**
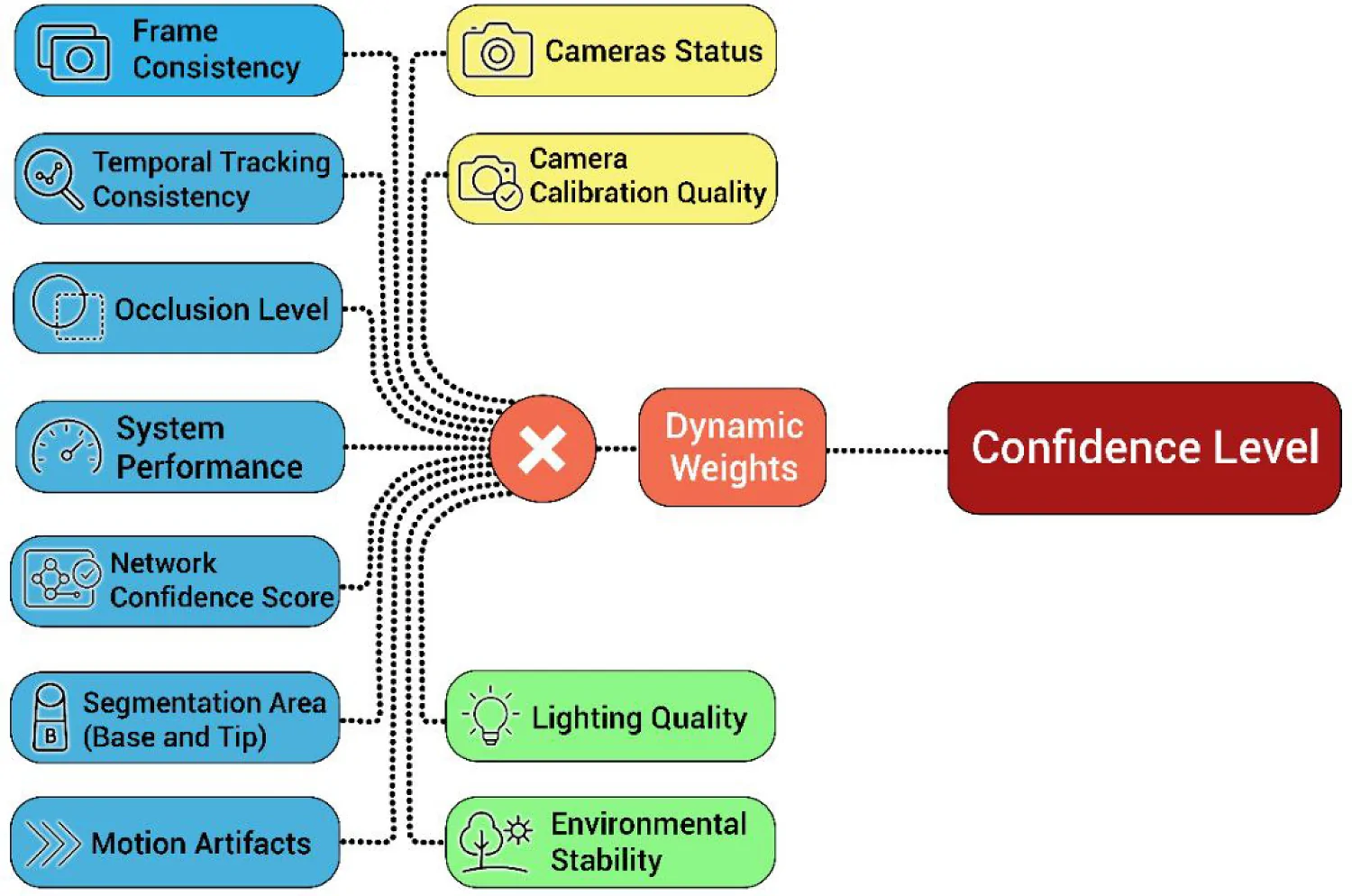
Effective metric categories

### Uncertainty metrics

Twelve metric categories capture complementary aspects of tracking quality (Table 1, Fig. 2). **Detection metrics** assess segmentation quality ($D_{\text {max}}$, $D_{\text {area}}$, $D_{\text {cons}}$); $D_{\text {max}}$ is defined as the mean of the maximum softmax probabilities across all camera views:

1 $$\begin{aligned} D_{\text {max}} = \frac{1}{K}\sum _{k=1}^{K} \max (\textrm{softmax}_k), \end{aligned}$$

where *K* is the number of active cameras. **Calibration quality** ($C_{\text {calib}}$) is derived from multi-view triangulation residuals; **camera validity** ($V_{\text {cam}}$) reflects the proportion of active views. **Environmental factors** capture lighting ($E_{\text {light}}$, mean V-channel luminance in HSV normalized to a calibration reference), occlusion ($E_{\text {occ}}$, ratio of observed to expected mask area), motion blur ($E_{\text {mot}}$, Laplacian variance of the grayscale ROI), and spatial stability ($E_{\text {stab}}$). The motion blur metric is defined as:

2 $$\begin{aligned} E_{\text {mot}} = \textrm{clip}\left( \frac{\textrm{Var}(\nabla ^2 I)}{\tau _{\text {mot}}},\, 0,\, 1\right) , \end{aligned}$$

where $\tau _{\text {mot}}$ is an empirically determined threshold and low variance indicates motion blur. All environmental pipelines are implemented in OpenCV. **Temporal metrics** assess trajectory smoothness ($T_{\text {temp}}$) and triangulation residuals ($T_{\text {res}}$). **System performance** ($S_{\text {perf}}$) monitors computational load, as resource saturation can cause frame drops and stale tracking outputs.

Note that $D_{\text {cons}}$ measures frame-to-frame stability of 2D segmentation outputs, whereas $T_{\text {temp}}$ operates on the final 3D triangulated positions and detects jumps in the fused trajectory that may arise even when individual 2D detections appear consistent.

All metrics are computed in their native units and normalized to [0, 100] using min-max normalization with empirically determined bounds (analytically derived for naturally bounded metrics; 1st/99th percentile clipping for unbounded metrics). Normalized metrics are fused through weighted aggregation to yield a reliability score R∈[0,100]. The reported uncertainty level is:

$$\begin{aligned} U = 100 - R. \end{aligned}$$

**Table 1:** Comprehensive definitions, value ranges, normalization strategies, and clinical justifications for all twelve uncertainty metrics

**Category**	**Metric**	**Computation rule**	**Raw range**	**Clinical justification**
Detection	$D_{\text {max}}$	Mean of max softmax probabilities across *K* camera views (Eq. 1)	[0, 1]	Quantifies segmentation model confidence; low values indicate uncertain detections
	$D_{\text {area}}$	Ratio of observed to expected mask area, normalized by reference	[0,∞)	Verifies physical plausibility of detected needle region
	$D_{\text {cons}}$	Frame-to-frame variation in 2D keypoint positions, normalized by estimated motion	[0,∞)	Detects erratic segmentation outputs across consecutive frames
Calibration	$C_{\text {calib}}$	RMS of multi-view reprojection residuals weighted by camera count	[0,∞) px	Monitors geometric accuracy of the multi-camera setup
Camera	$V_{\text {cam}}$	Proportion of active cameras contributing to triangulation	[0, 1]	Fewer views reduce triangulation robustness
Environment	$E_{\text {light}}$	Mean V-channel luminance in HSV, normalized against calibration reference	[0, 1]	Severe illumination changes cause segmentation failure
	$E_{\text {occ}}$	Ratio of observed to expected segmentation mask area from calibrated model	[0, 1]	Occlusion removes visual evidence for localization
	$E_{\text {mot}}$	Laplacian variance of grayscale ROI, clipped and normalized (Eq. 2)	[0, 1]	Motion blur degrades feature extraction quality
	$E_{\text {stab}}$	Spatial drift of needle position relative to expected trajectory	[0,∞) mm	Detects gradual positional drift over time
Temporal	$T_{\text {temp}}$	Smoothness of 3D trajectory over a sliding window (std. dev. of positions)	[0,∞) mm	Detects sudden jumps in fused 3D output
	$T_{\text {res}}$	Triangulation residual magnitude across camera pairs	[0,∞) px	High residuals indicate geometric inconsistency
System	$S_{\text {perf}}$	Weighted combination of CPU load, memory usage, and disk I/O utilization	[0, 1]	Resource saturation causes frame drops and stale outputs

### Uncertainty computation methods

#### Classic method

The classic method computes an overall reliability score (*R*) as a weighted sum of interpretable sub-metrics:

3 $$\begin{aligned} R =\;&w_1 D_{\text {max}} + w_2 D_{\text {area}} + w_3 D_{\text {cons}} + w_4 C_{\text {calib}} + w_5 V_{\text {cam}} \nonumber \\&+ w_6 E_{\text {light}} + w_7 E_{\text {occ}} + w_8 E_{\text {mot}} + w_9 E_{\text {stab}} \nonumber \\&+ w_{10} T_{\text {temp}} + w_{11} T_{\text {res}} + w_{12} S_{\text {perf}} \end{aligned}$$

where $w_1,\ldots ,w_{12}$ are nonnegative base weights subject to $\sum _{i=1}^{12} w_i = 1$, heuristically initialized and dynamically adjusted in real time (e.g., increasing $w_9$ when $E_{\text {stab}}$ indicates drift) while maintaining normalization. When a metric crosses an empirically determined degradation threshold, its corresponding weight is multiplied by a predefined scaling factor greater than 1, and all weights are immediately renormalized to satisfy $\sum w_i = 1$. Thresholds and scaling factors were determined empirically via a grid search on the training partition, selecting values that maximized the Pearson correlation between predicted uncertainty and ground-truth tracking error. The pipeline computes sub-metrics as defined in the Uncertainty metrics Section (Table 1), fuses them with dynamic weights, and reports U=100-R. A fail-safe suppresses spuriously low values under degraded conditions.

The processing applies three sequential stages: (1) dynamic weight adjustment with re-normalization, (2) degradation penalty computation via Eq. 4, and (3) exponential moving average smoothing via Eq. 5. These mechanisms are complementary: the penalty adjusts instantaneous uncertainty magnitude under catastrophic conditions, while the EMA smooths the temporal trajectory.

Robustness is improved via an adaptive fail-safe mechanism. A smooth degradation penalty P∈[0,1] is computed as:

4 $$\begin{aligned} P = \max \left( \sigma \left( \frac{0.5 - E_{\text {light}}}{0.1}\right) , \sigma \left( \frac{E_{\text {occ}} - 0.5}{0.1}\right) , \sigma \left( \frac{70 - T_{\text {res}}}{10}\right) \right) \nonumber \\ \end{aligned}$$

where $$\sigma (x) = 1/(1+e^{-x})$$ ensures continuous transitions and $$E_{\text {light}}$$, $$E_{\text {occ}}$$, and $$T_{\text {res}}$$ are computed as specified in the Section Uncertainty metrics and Table 1. The three metrics were selected because they capture catastrophic failure modes—complete loss of illumination, visual evidence, or geometric consistency—rather than gradual degradation. The max operator ensures that any single critically degraded condition triggers the penalty, since each failure alone can render tracking unreliable. The final uncertainty is adjusted as $U^{\prime }=U+P\cdot \left(100-U\right)$, proportionally boosting uncertainty based on degradation severity. This complements the weighted aggregation in *R*, which captures gradual multi-factor degradation.

To prevent rapid oscillations while maintaining responsiveness, temporal smoothing is applied via an exponential moving average:

5 $$\begin{aligned} U_t = \alpha \cdot U'_t + (1-\alpha ) \cdot U_{t-1} \end{aligned}$$

where α=0.7 balances noise suppression and adaptive response. The classic method is deep-learning-independent and real time (<5 ms per frame), suitable for constrained hardware and safety-critical deployment.

#### Hybrid CNN method

**Fig. 3:**
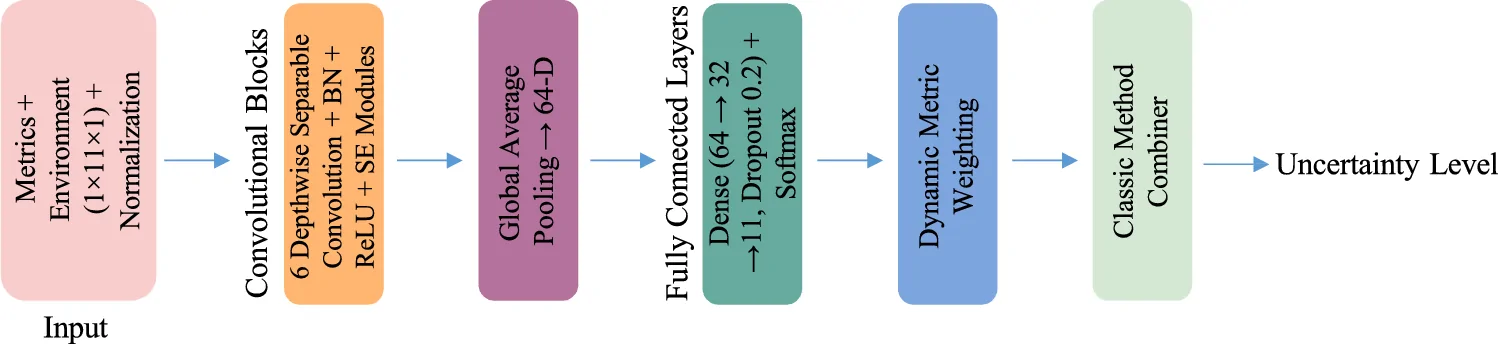
Hybrid CNN architecture

To combine the interpretability of the classic method with the adaptability of deep learning, we propose a hybrid approach in which a CNN dynamically predicts the metric weights $(w_1,\ldots ,w_{11})$. Based on preliminary weight-optimization experiments, we adopt a lightweight architecture inspired by MobileNetV3-Small [15], chosen for its favorable accuracy–efficiency trade-off, enabling real-time inference on clinical hardware. The hybrid CNN predicts weights for 11 metrics, excluding $S_{\text {perf}}$, which contributes a fixed penalty independent of visual input to avoid hardware-specific overfitting.

The network takes an 11-dimensional feature vector of normalized metric values in [0, 1]. Environmental metrics ($E_{\text {light}}$, $E_{\text {occ}}$, $E_{\text {mot}}$) serve as preprocessed contextual inputs rather than learned weights to prevent circular dependency during optimization: The network would otherwise need to simultaneously estimate the environmental condition and decide its influence on the final uncertainty, which empirically led to unstable training. These environmental cues are computed via OpenCV pipelines as defined in the Section Uncertainty metrics.

The architecture comprises six convolutional blocks with depthwise separable convolutions, followed by global average pooling to produce a 64-dimensional representation. Two fully connected layers with dropout (p=0.2) map this to 11 softmax-normalized weights (Fig. 3), which are combined with the original metrics via the classic method to produce U=100-R.

Training used 25,000 frames with Adam (lr $= 5\times 10^{-4}$, batch size 32), MSE loss against ground-truth uncertainty targets $U_{\text {gt}} = \textrm{clip}(100 \cdot E/10,\, 0,\, 100)$, up to 50 epochs with early stopping (patience =8) and learning rate reduction on plateau (factor 0.5, patience =3).

#### Full CNN method

**Fig. 4:**
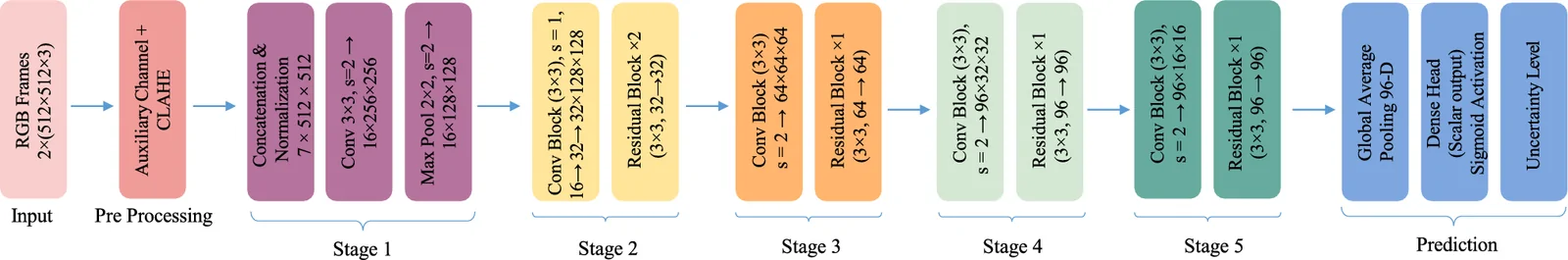
Full CNN Architecture

The full CNN employs a compact architecture (∼500 K parameters) to estimate uncertainty directly from raw frames (Fig. 4). The network takes a multi-channel input formed by two synchronized RGB frames resized to 512×512, concatenated along the channel dimension, and augmented with an auxiliary channel encoding lighting and occlusion cues preprocessed with OpenCV.

The backbone is a streamlined residual-style feature extractor with lightweight bottleneck blocks using 3×3 convolutions, batch normalization, ReLU activations, and skip connections. Global average pooling produces a compact latent vector, which a fully connected head maps to a single scalar. A sigmoid activation bounds the output to [0, 1], scaled to $U \in [0,100]\%$. The training target is derived from the ground-truth tracking error *E*, computed as the Euclidean distance between the tracking system prediction and the independent reference measurement (Datasets Section), and mapped to an uncertainty target via the linear calibration defined in the Uncertainty-to-error calibration Section:

$$\begin{aligned} U = \textrm{clip}\left( 100 \cdot \frac{E}{10},\, 0,\, 100\right) . \end{aligned}$$

Training used Adam (learning rate $1\times 10^{-4}$, batch size 16, up to 80 epochs) with early stopping on validation loss.

### Uncertainty-to-error calibration

This section defines a fixed, interpretable linear mapping between predicted uncertainty and spatial tracking error.

The uncertainty level $U \in [0,100]\%$ is linked to tracking error *E* (in millimeters) through

$$\begin{aligned} U = \textrm{clip}\left( 100 \cdot \frac{E}{10},\, 0,\, 100\right) , \end{aligned}$$

such that U=0% corresponds to ideal tracking (E=0 mm) and U=100% corresponds to E=10 mm. In deployment, an estimated error can be displayed for user feedback using

$$\begin{aligned} {\hat{E}} = \frac{U}{100} \cdot 10\,\text {mm}. \end{aligned}$$

This formulation removes the need for heuristic threshold tables and provides a consistent interpretation of uncertainty across methods, datasets, and operating conditions. The 10-mm upper bound reflects two considerations. First, clinically acceptable tracking errors are application-dependent, ranging from sub-2 mm in neurosurgical navigation to 9±6 mm in electromagnetic navigation bronchoscopy  [16, 17] and 10.1±4.0 mm in freehand CT-guided liver ablation [18]. Second, tracking error is only one component of the total error chain, alongside needle deflection, respiratory motion (up to 17.5 mm [19]), and CT-to-body divergence [20]. To validate this mapping, uncertainty–error pairs were computed for each frame and pooled for statistical analysis.

## Results

### Evaluation metrics

The primary measure is the tracking error, computed as the Euclidean distance between predicted and ground-truth 3D coordinates:

$$\begin{aligned} \text {Error} = \sqrt{(x_{\text {pred}} - x_{\text {GT}})^2 + (y_{\text {pred}} - y_{\text {GT}})^2 + (z_{\text {pred}} - z_{\text {GT}})^2} \end{aligned}$$

The Pearson correlation coefficient (*r*) measures the linear correlation between uncertainty (*U*) and tracking error (*E*):

$$\begin{aligned} r = \frac{\sum _{i=1}^{n} (U_i - {\bar{U}})(E_i - {\bar{E}})}{\sqrt{\sum _{i=1}^{n} (U_i - {\bar{U}})^2 \sum _{i=1}^{n} (E_i - {\bar{E}})^2}} \end{aligned}$$

The mean absolute error (MAE) quantifies the average difference between the error implied by uncertainty (${\hat{E}}=\frac{U}{100}\cdot 10$ mm) and the ground-truth tracking error:

$$\begin{aligned} \text {MAE} = \frac{1}{n} \sum _{i=1}^{n} |{\hat{E}}_{i} - E_{i}| \end{aligned}$$

Computational efficiency is measured by frame processing time.

### Validation of uncertainty-to-error correlation

A strong positive correlation between uncertainty and tracking error was consistently observed across all datasets and methods (Table 2), confirming that higher uncertainty reliably corresponds to larger spatial errors and validating the framework’s core hypothesis.

**Table 2:** Statistical validation: Pearson correlation Coefficient (*r*)

**Dataset**	**Classic method**	**Hybrid CNN**	**Full CNN**
Semi-clinical	0.84	0.83	0.85
Clinical	0.87	0.84	0.82

### Dynamic system performance

**Fig. 5:**
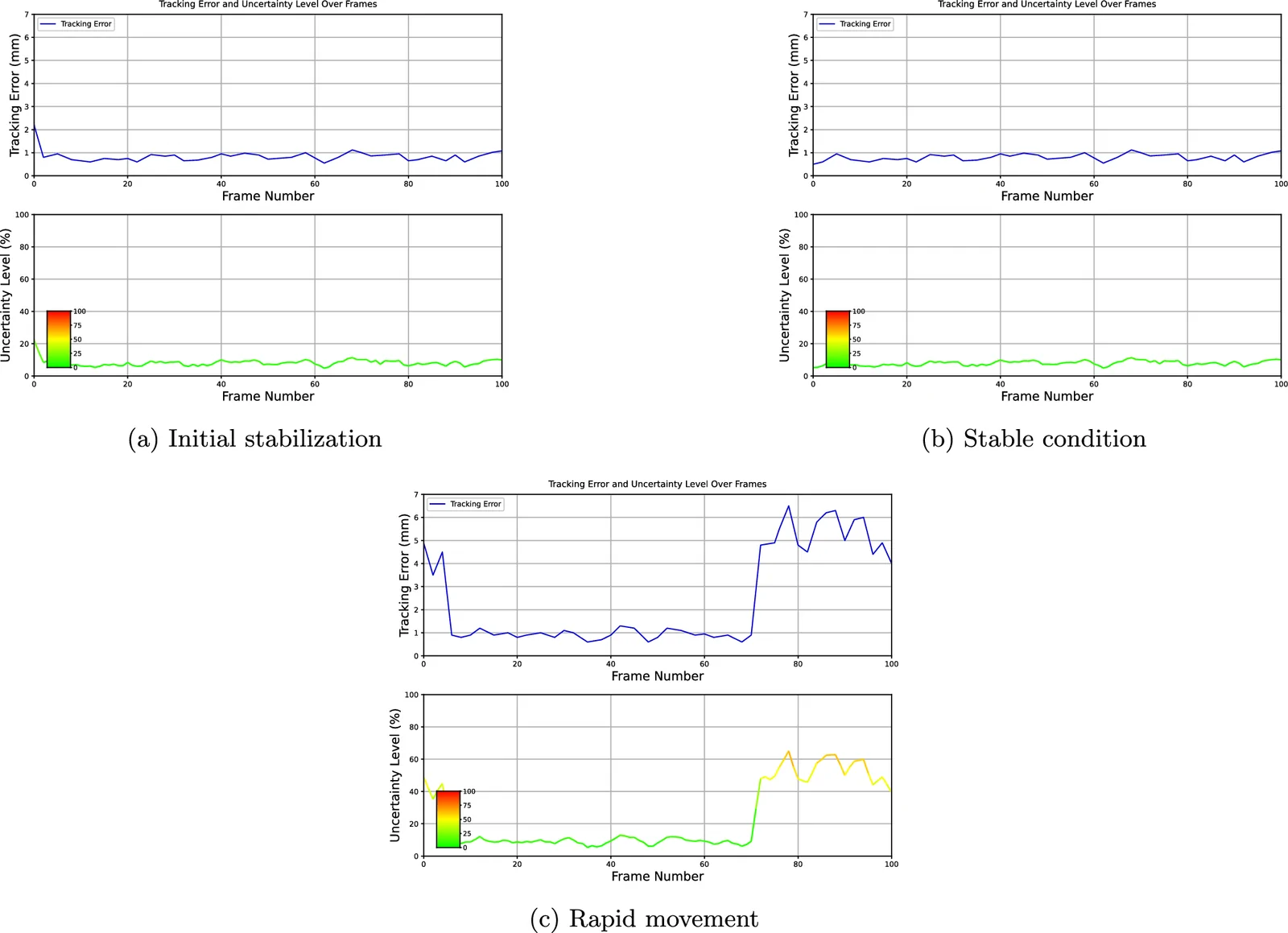
Relationship between tracking error and uncertainty level over time under different conditions

The dynamic relationship between tracking error and uncertainty is shown in Fig. 5. During initial needle instability, the framework assigned high uncertainty that decreased as the error converged to a sub-millimeter baseline (Fig. 5a). Under stable conditions, uncertainty remained low (<10%) with consistently low error (Fig. 5b). For rapid displacements, uncertainty increased proportionally and recovered promptly once motion ceased (Fig. 5c). Method comparison (Table 3) revealed complementary trade-offs: full CNN achieved the highest accuracy (MAE = 0.48 mm, 7.2 ms), classic near-optimal accuracy with fastest inference (0.53 mm, 5.1 ms) and full interpretability, and hybrid CNN (0.54 mm, 9.7 ms) balanced learned adaptivity with metric transparency. All methods achieve real-time performance.

**Table 3:** Uncertainty computation methods on the semi-clinical dataset

**Method**	**MAE (mm)**	**Processing time (ms)**	**Parameters (M)**
Classic	0.53	5.1	N/A
Full CNN	0.48	7.2	0.48
Hybrid CNN	0.54	9.7	0.5

### Scenario-based evaluation and output illustration

**Fig. 6:**
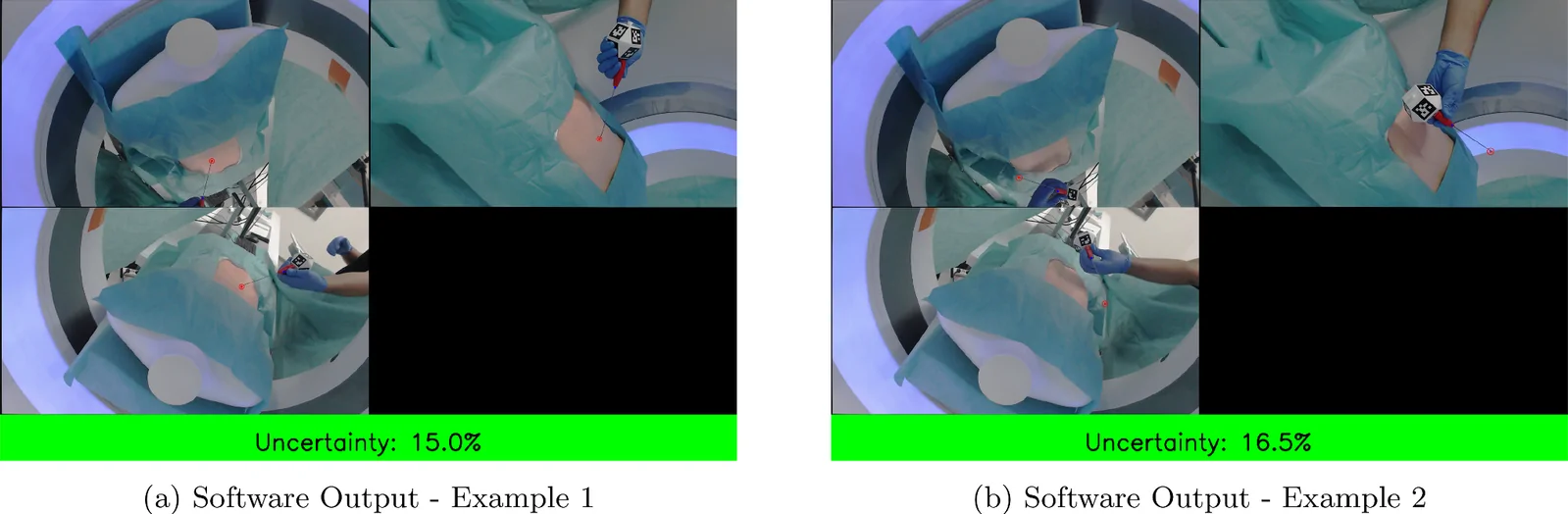
Software output under stable conditions

**Fig. 7:**
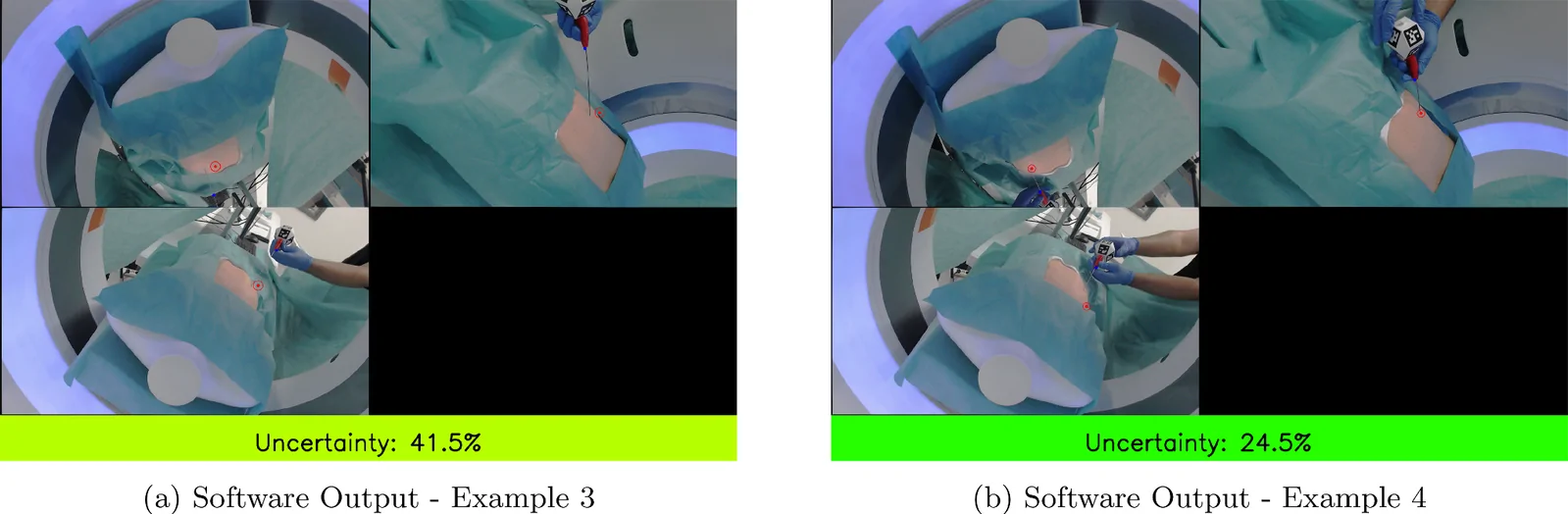
Software output under tracking deterioration and recovery

**Fig. 8:**
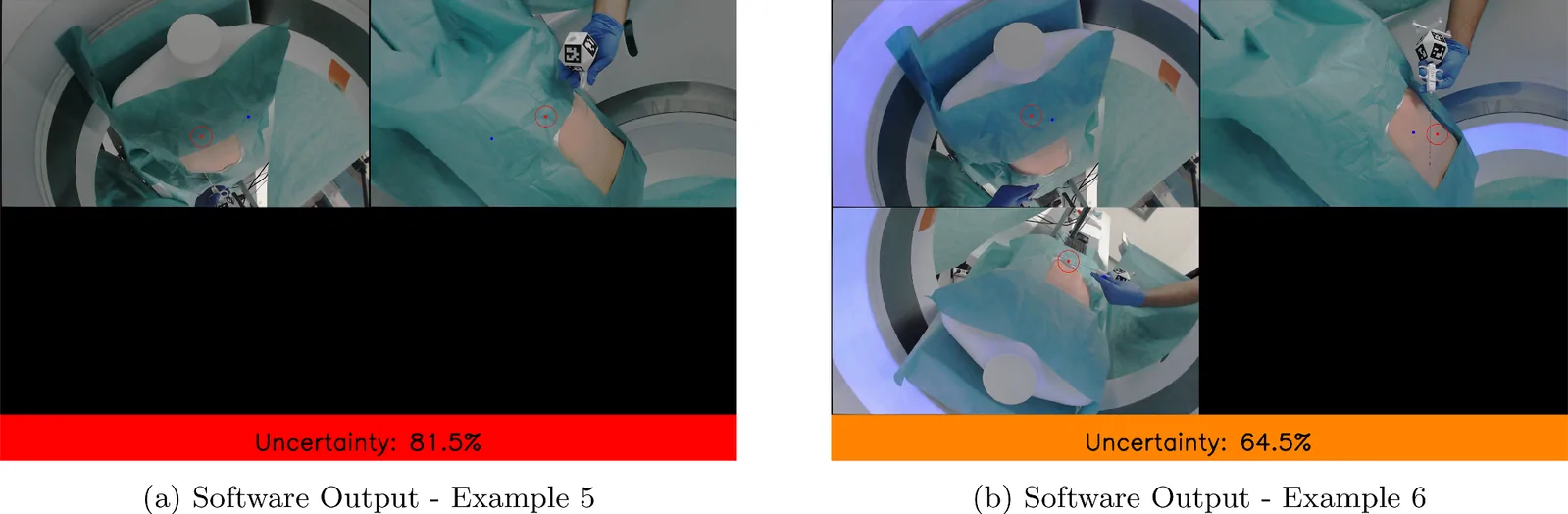
Software output under rapid motion and camera occlusion

**Fig. 9:**
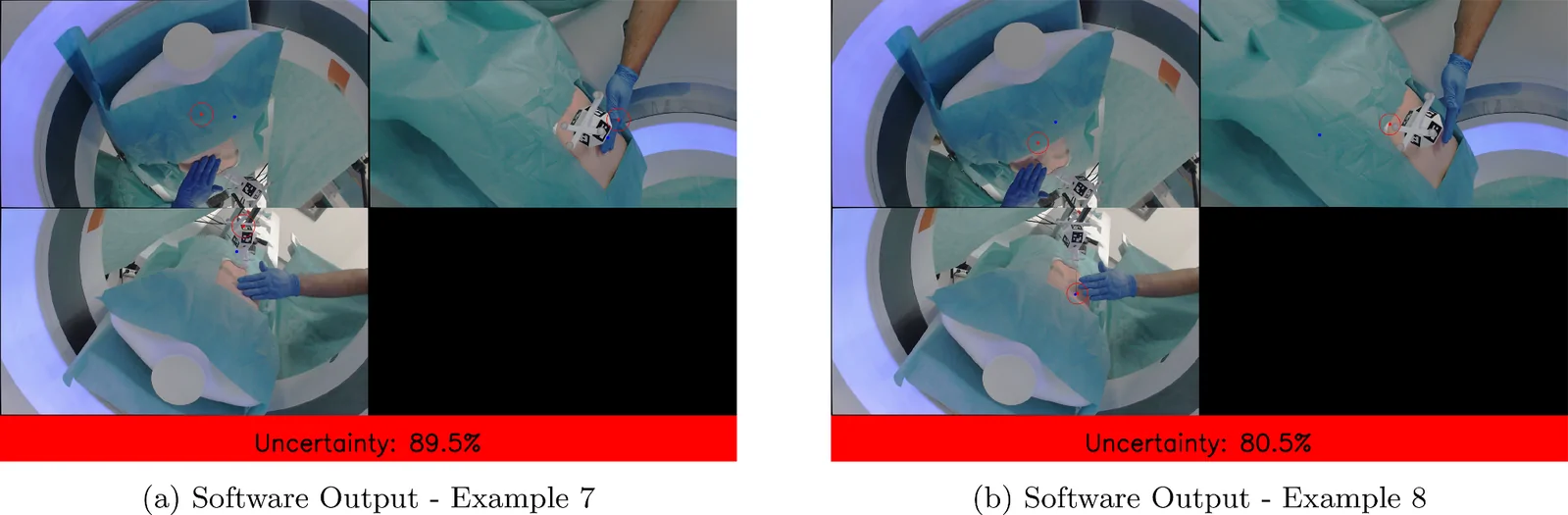
Software output under full tool occlusion and tracking failure

Representative results from four scenarios are presented using the full CNN method (Figs. 6, 7,  8, and 9). The GUI displays tracked needle overlays, uncertainty levels, a dynamic color-encoded bar, and a secondary ring whose diameter scales with uncertainty. Additional results and demonstration videos are provided in the Supplementary Material.

#### Scenario 1: stable conditions

Low uncertainty (<17%) with errors <1.7 mm confirmed reliable performance under stable conditions (Fig. 6).

#### Scenario 2: tracking deterioration and recovery

Tracking deterioration increased uncertainty to >40% and introduced errors >4 mm. During recovery, uncertainty decreased gradually despite persistent degradation in one camera view (Fig. 7ba).

#### Scenario 3: rapid motion and camera occlusion

Rapid motion and camera occlusion increased uncertainty to >80% and led to peak errors >10 mm, demonstrating the correct detection of tracking failure. The system recovered autonomously as conditions improved (Fig. 8).

#### Scenario 4: full tool occlusion and tracking failure

Complete instrument occlusion raised uncertainty to >80% with errors exceeding 10 mm. This reveals a limitation: under total visual obstruction, the framework occasionally reports moderate uncertainty even in the absence of valid input (Fig. 9).

## Discussion

The method comparison revealed complementary strengths. The full CNN achieved the best accuracy (MAE = 0.48 mm) with real-time performance (7.2 ms), demonstrating the advantages of end-to-end learning under complex degradations. Classic achieved near-optimal accuracy (MAE = 0.53 mm) with the fastest inference (<5.1 ms) and full interpretability, making it ideal for resource-constrained or safety-critical deployments. Hybrid CNN (MAE = 0.54 mm, 9.7 ms) offered a middle ground, preserving metric transparency while adding learned adaptivity.

Limitations remain: (1) single-site, phantom-only evaluation without in vivo or multicenter testing; (2) fixed linear 0–10-mm calibration that may not optimally reflect error distributions across diverse procedural contexts; (3) global uncertainty without spatial differentiation (e.g., tip-specific vs. base-specific); (4) absence of clinical validation with surgeons to assess interpretability and trust; (5) limited analysis of false-confidence scenarios (see Supplementary Material); and (6) uncertain generalization across anatomical regions and CT-lab environments.

To address these limitations, we are conducting user studies with interventional radiologists assessing workflow integration and trust. Future work will extend validation to diverse settings, investigate spatial uncertainty differentiation, and rare failure modes under extreme degradations.

## Conclusion

This study introduces a dynamic uncertainty-assessment framework for real-time needle tracking in CT-guided interventions. By establishing a validated linear relationship between uncertainty levels and physical tracking errors, the framework translates abstract reliability into a clinically actionable metric, enhancing procedural safety and operator trust.

All three approaches achieve real-time performance with complementary trade-offs: full CNN provides the highest accuracy, classic offers optimal efficiency and interpretability, and Hybrid CNN balances adaptability with transparency. We are actively pursuing clinical validation with interventional radiologists. Future work will focus on embedding this framework into closed-loop surgical systems with intra-instrument and visual feedback.

## Supplementary Information

Below is the link to the electronic supplementary material.

Supplementary file 1 (pdf 31186 KB)

Supplementary file 2 (mp4 7440 KB)
