# Supplementary material for: Dynamic uncertainty-level assessment framework for real-time needle tracking in CT-guided surgical environments
Source: Int J Comput Assist Radiol Surg. 2026 Jun 5;21(10):2135–45. doi: 10.1007/s11548-026-03725-w (PMC13723632; doi:10.1007/s11548-026-03725-w)
Supplement: Supplementary file 1 [file 11548_2026_3725_MOESM1_ESM.pdf]

Supplementary Material for *'Dynamic Uncertainty Level Assessment Framework for Real-Time Needle Tracking in CT-Guided Surgical Environments'*

Max Steiger[1], Mohammad Rezapourian[1], Marko Rak[1], and Christian Hansen[1]

[1]Research Campus STIMULATE, University of Magdeburg, Otto-Hahn-Straße 2, 39106 Magdeburg, Saxony-Anhalt, Germany

CARS 2026 Computer Assisted Radiology and Surgery

S1. Additional Software Interface Examples

S1.1 Low uncertainty scenarios

The following two examples demonstrate stable tracking with acceptable uncertainty during normal needle movement.

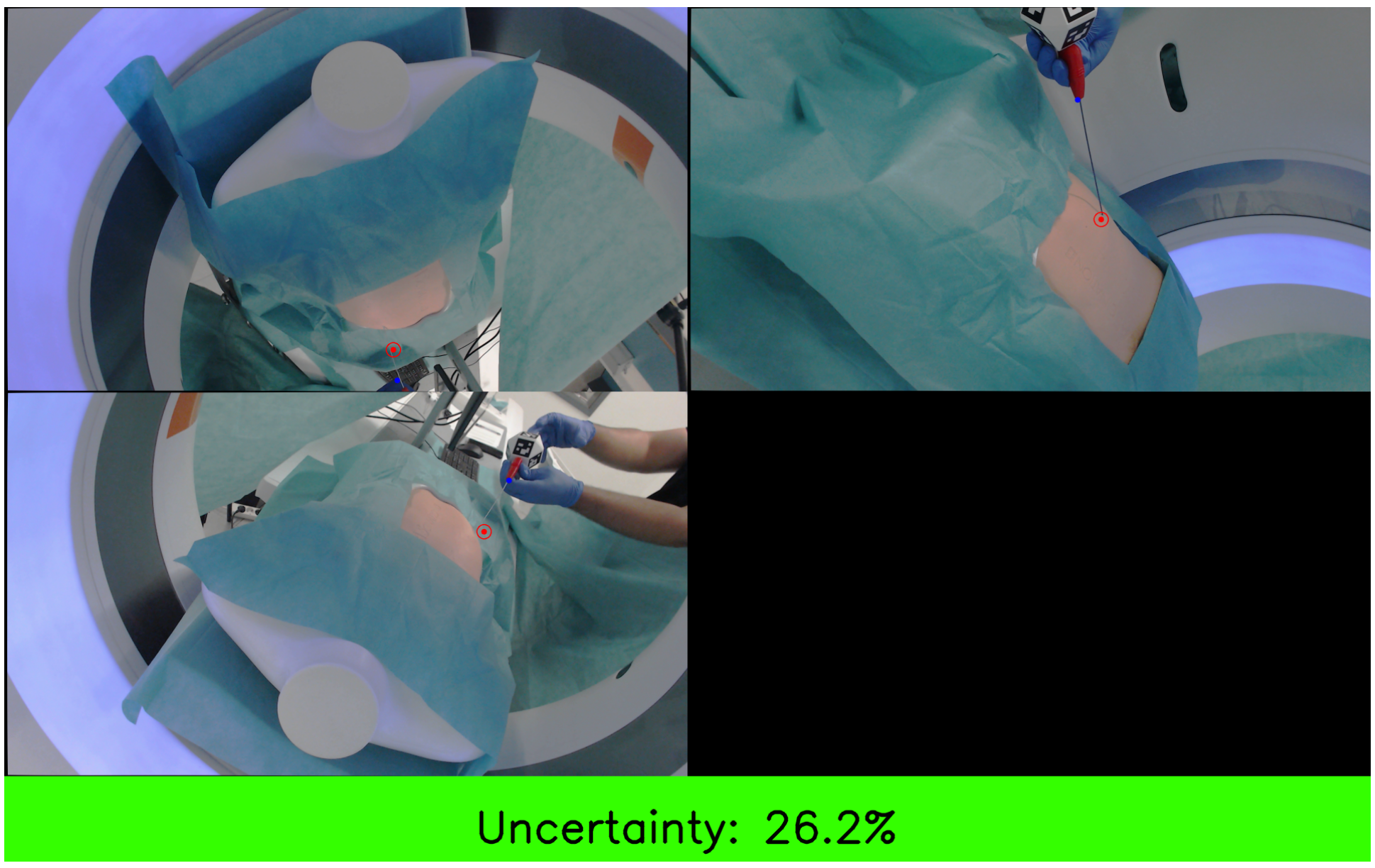

Figure 1: Software Interface view showing a low uncertainty case (Example 1).

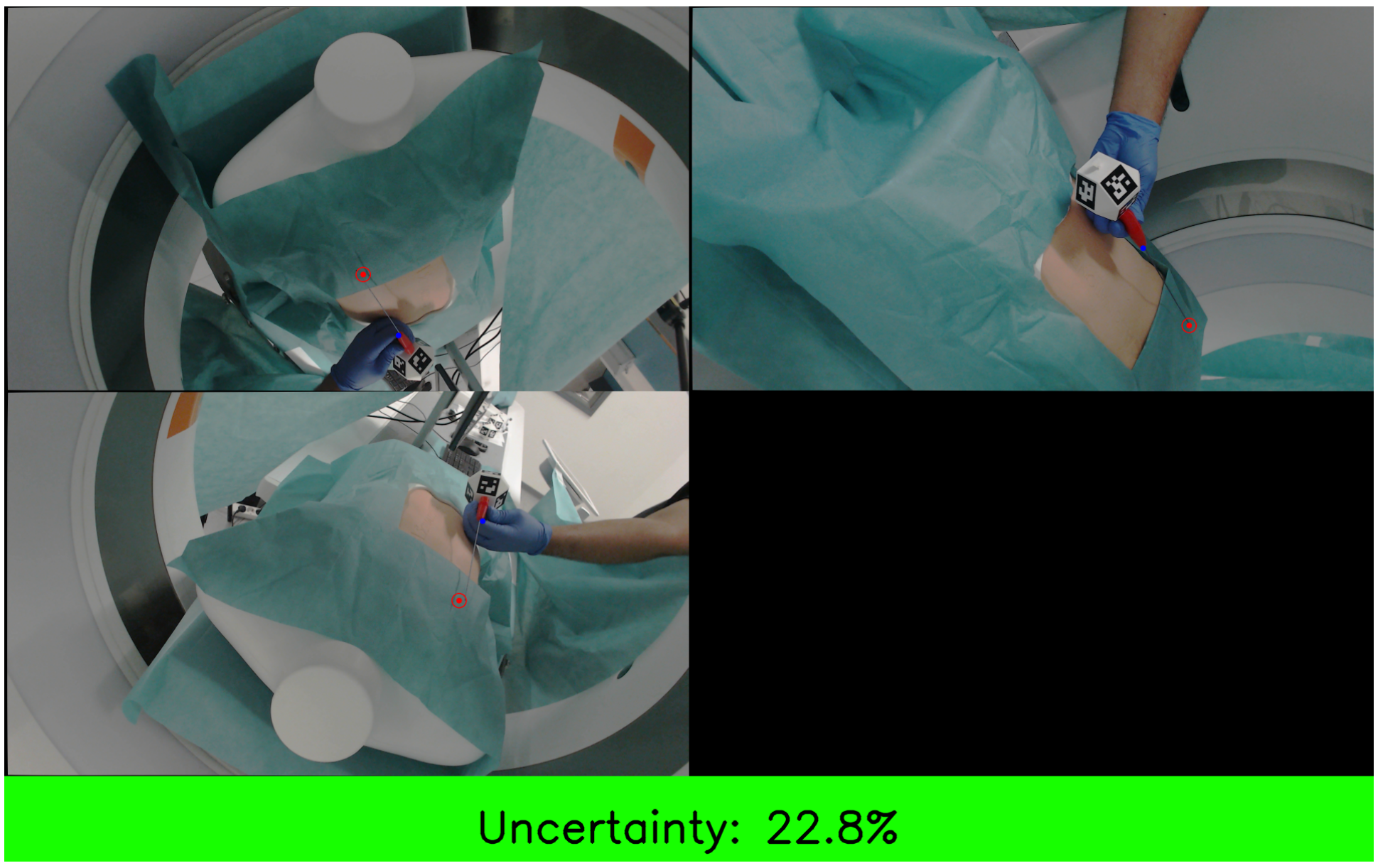

Figure 2: Software Interface view showing a low uncertainty case (Example 2).

S1.2 Moderate uncertainty scenarios

With tracking loss in one camera frame (Figure 3), the uncertainty rises but still remains in a usable area. As tracking degrades further, uncertainty increases, and the visual cue ring enlarges. Tracking with moderate uncertainty can still be used, as at least one camera maintains a stable lock (Figure 4). It is up to the user to determine whether the risk remains acceptable.

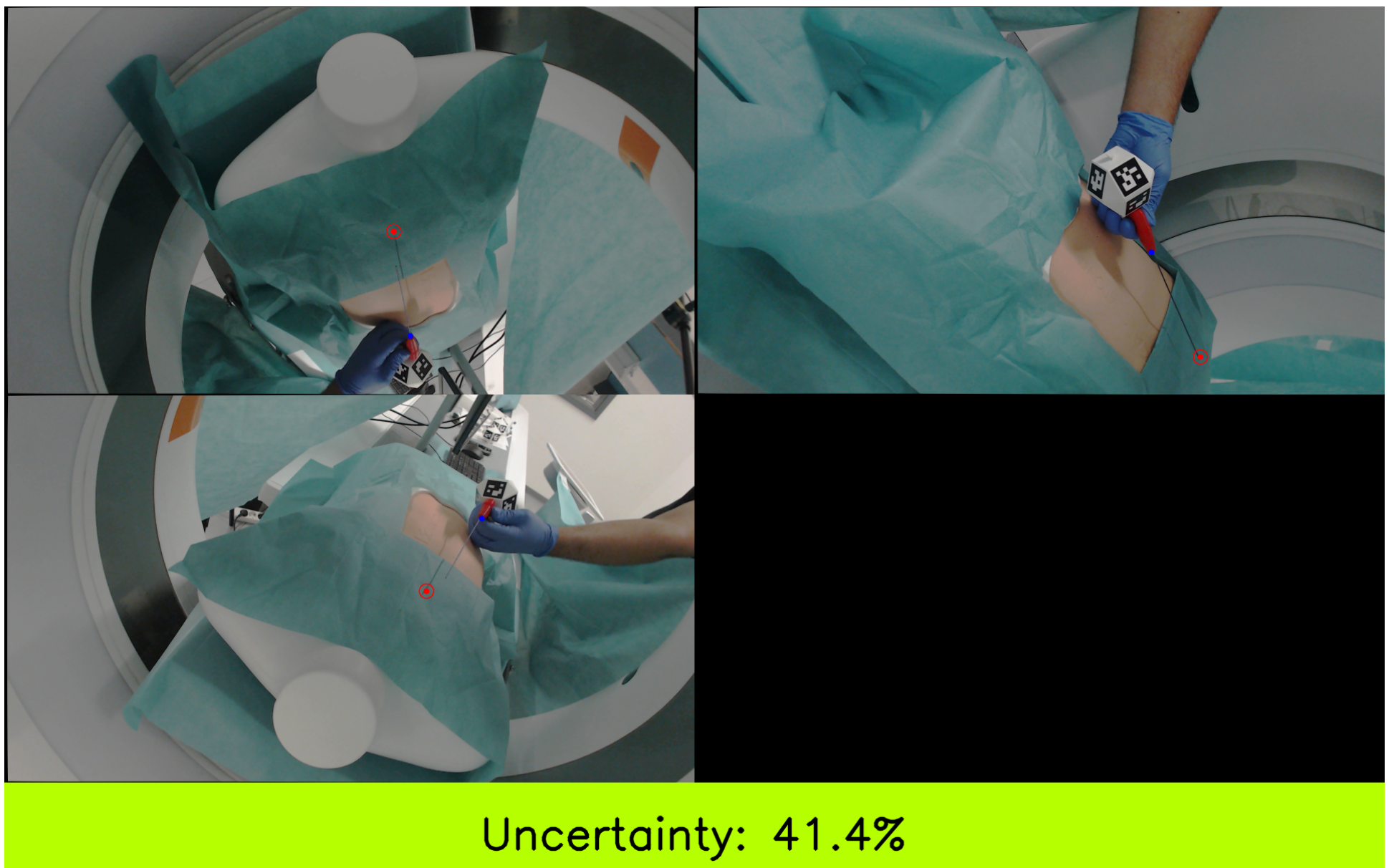

Figure 3: Software Interface view showing a moderate uncertainty case (Example 1).

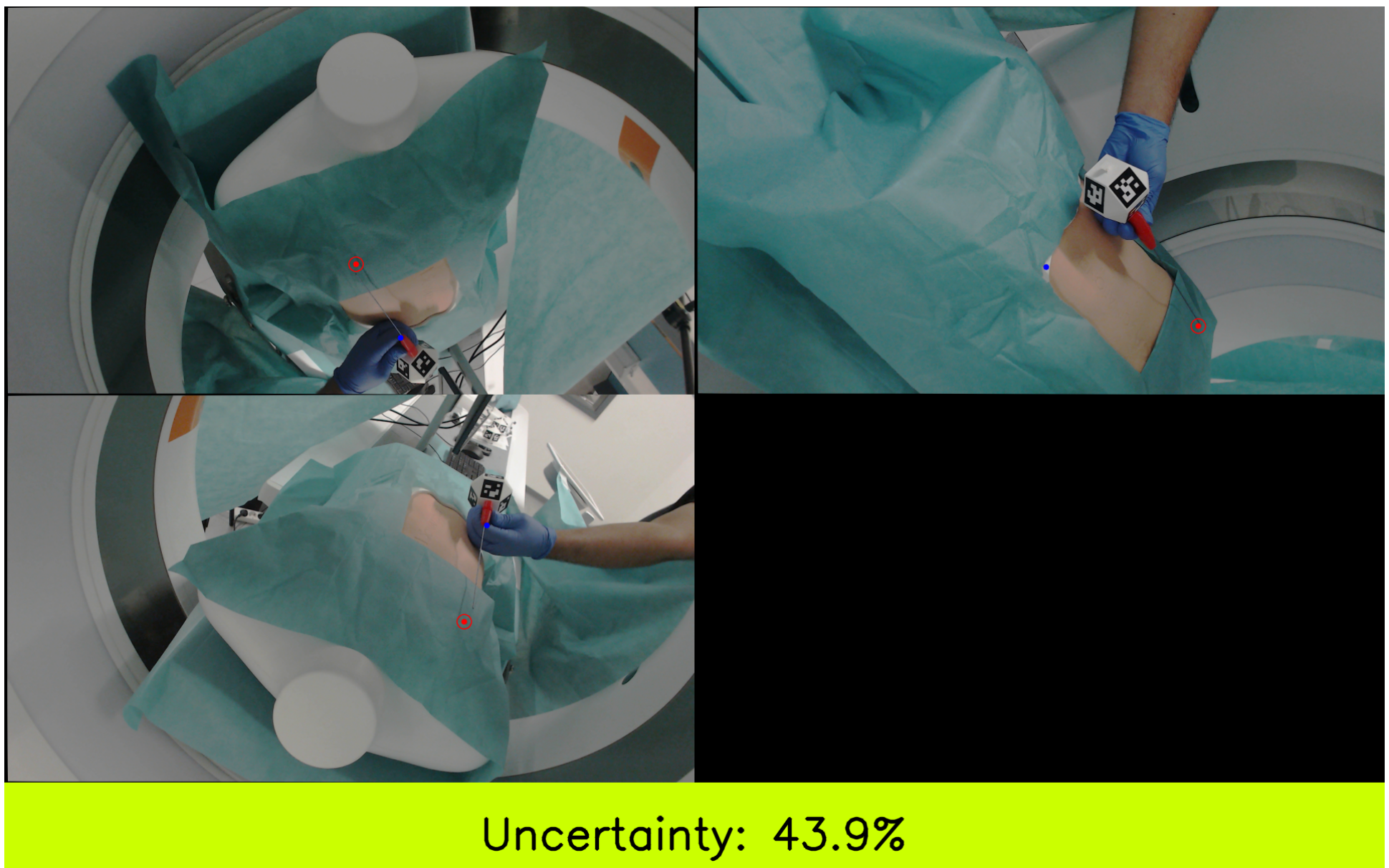

Figure 4: Software interface view showing a moderate uncertainty case (Example 2).

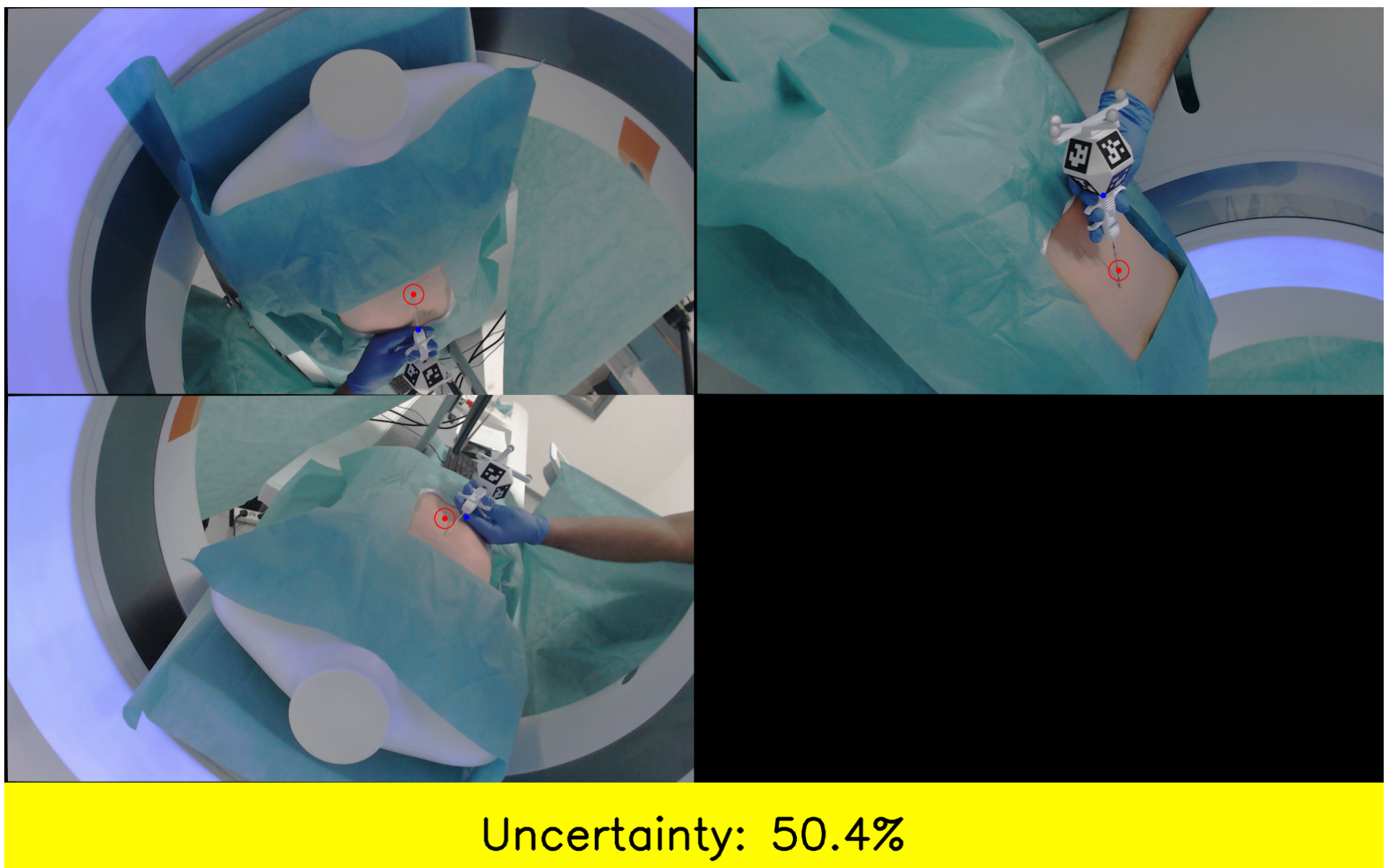

Figure 5: Software interface view showing a moderate uncertainty case (Example 3).

S1.3 High uncertainty scenarios & Failure

In these cases, our system either partially failed or exhibited high uncertainty, as expected due to tracking inaccuracies or failures. In the first case (Figure 6), our system does not reach the maximum uncertainty, even when all tracking is lost (except for a still highly inaccurate tracking in one camera). In the second case, the tracking shows substantial variation in needle-tip predictions across cameras, with only one camera yielding an accurate result. This yields the desired high uncertainty, which predicts the correct average tracking error across the 3 camera views.

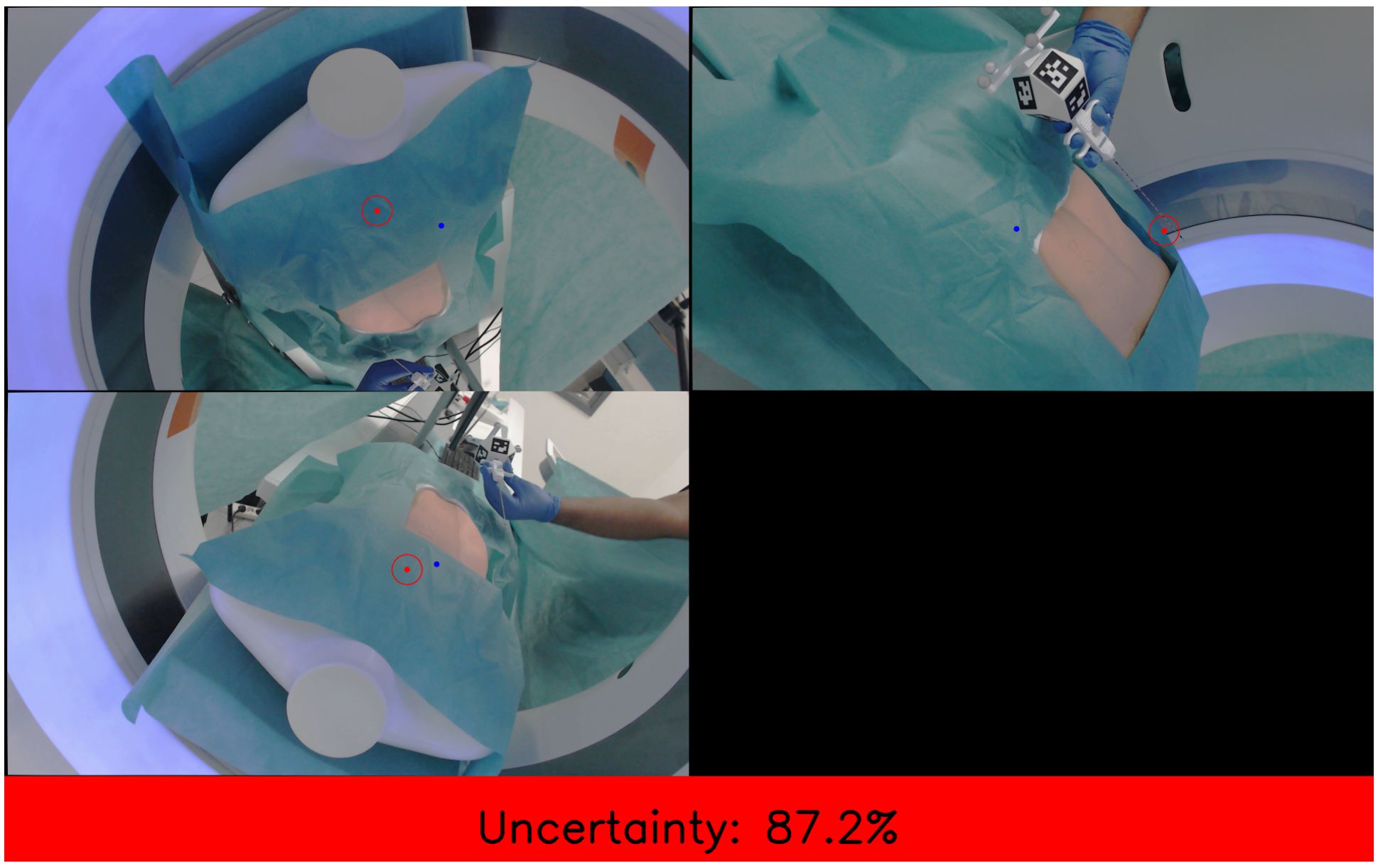

Figure 6: Software Interface view showing a high uncertainty case (Example 1).

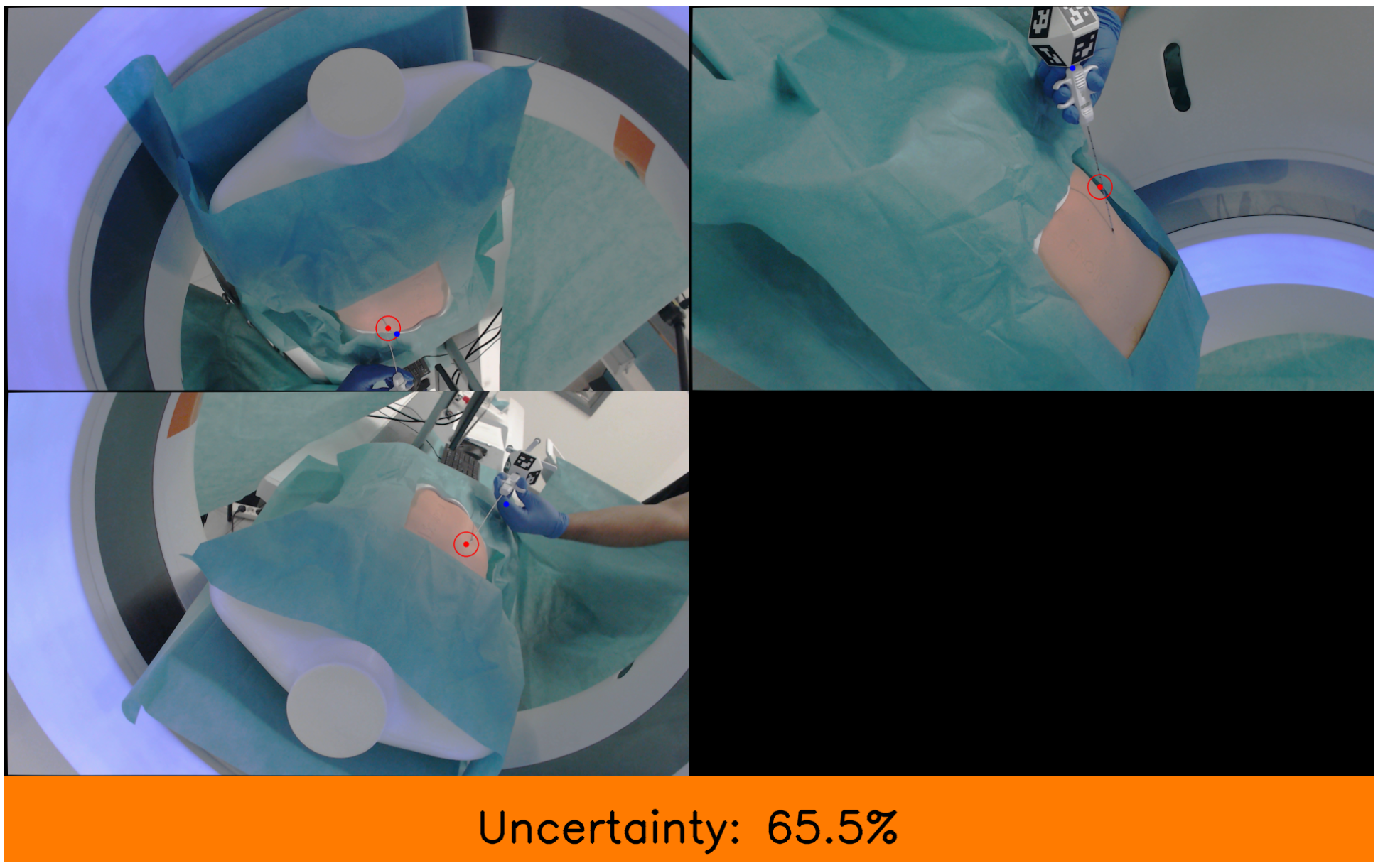

Figure 7: Software interface view showing a high uncertainty case (Example 2).

S2. Extended Quantitative Results

The following plots present additional dynamic behavior of our dynamic uncertainty estimation framework. We observe stable tracking dynamics and rapid needle movements, with tracking error that varies and results in higher uncertainty.

S2.1 Stable Condition Performance

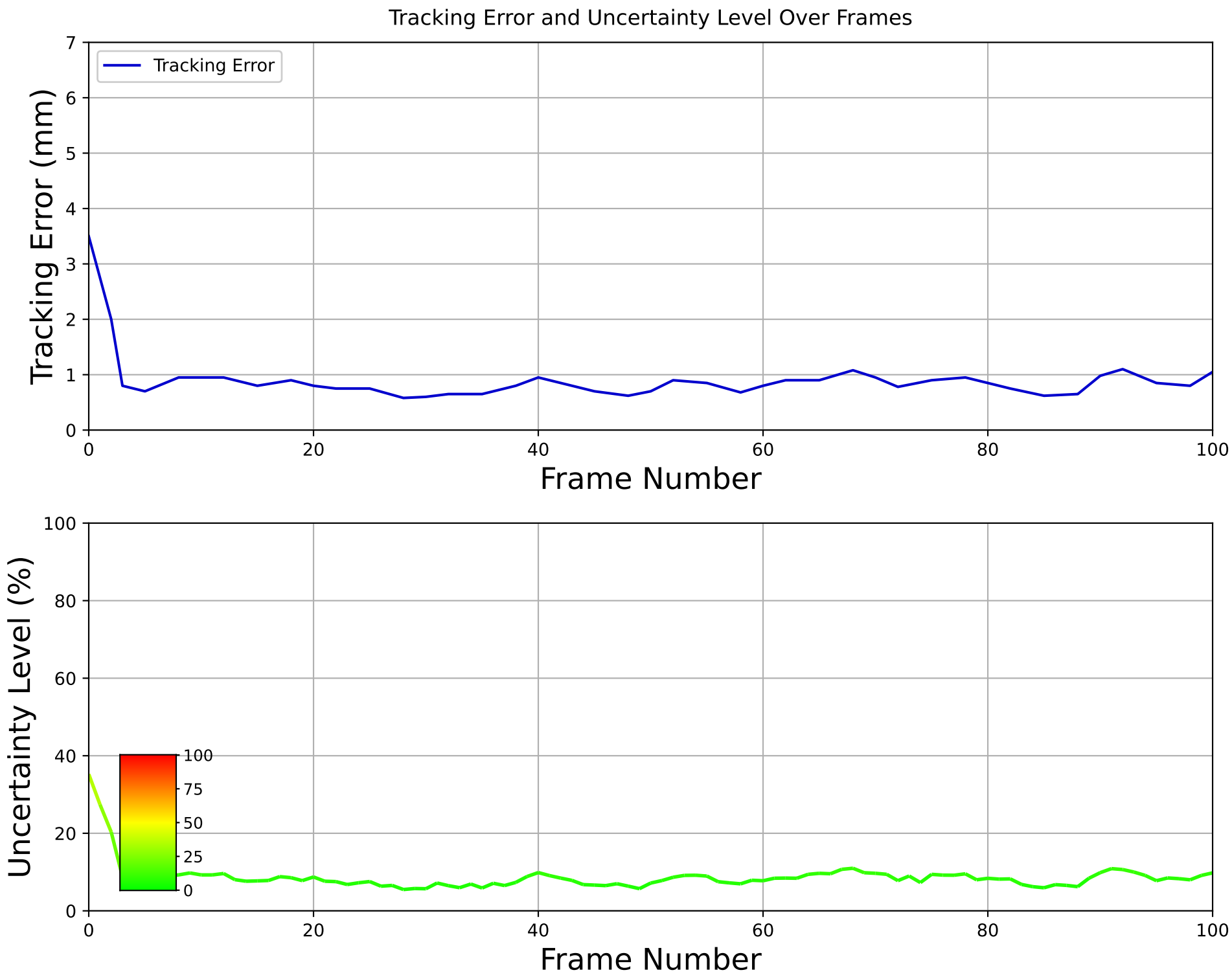

Figure 8: Result plot for a stable condition on clinical dataset (Example 1).

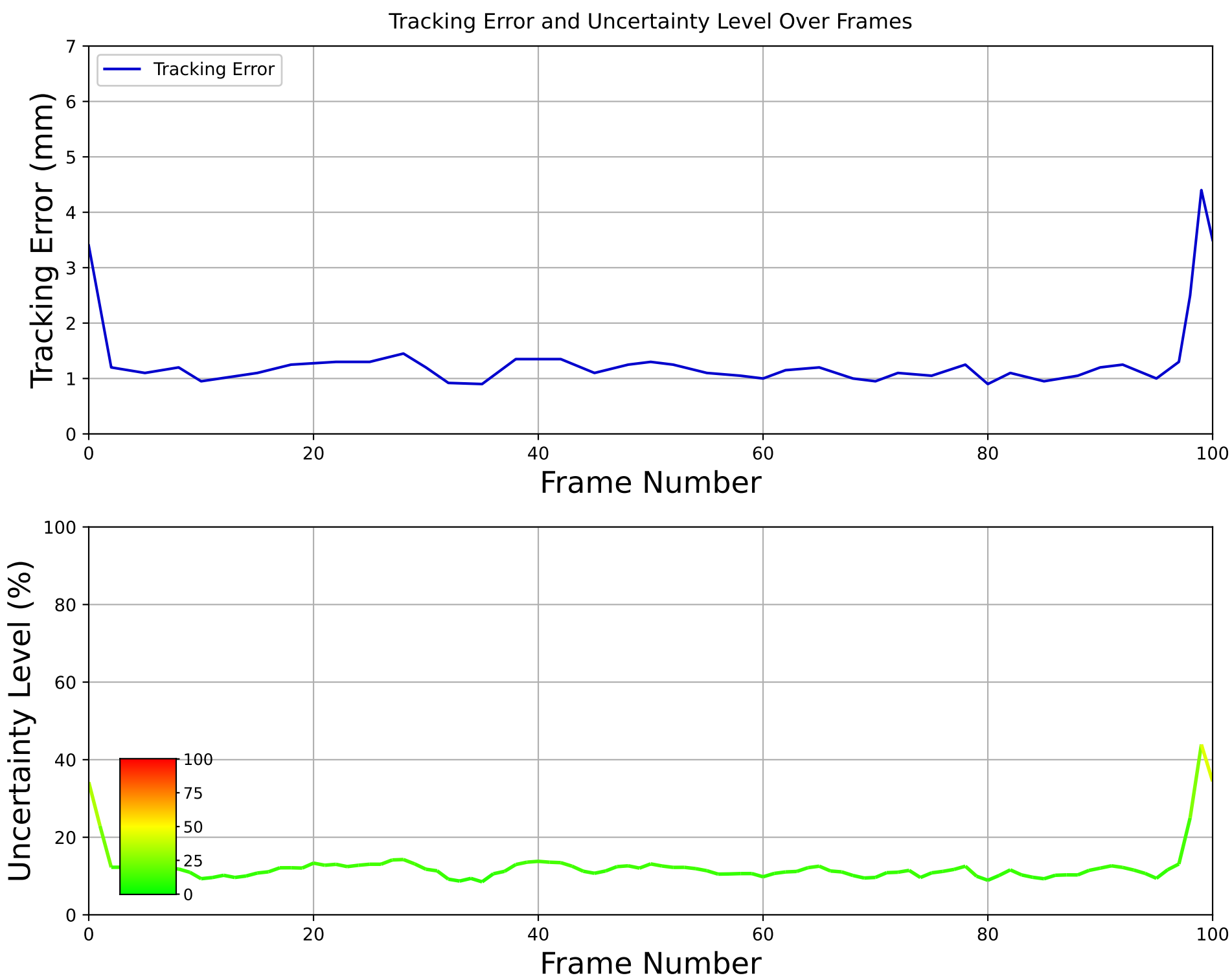

Figure 9: Result plot for a stable condition on semi-clinical dataset (Example 2).

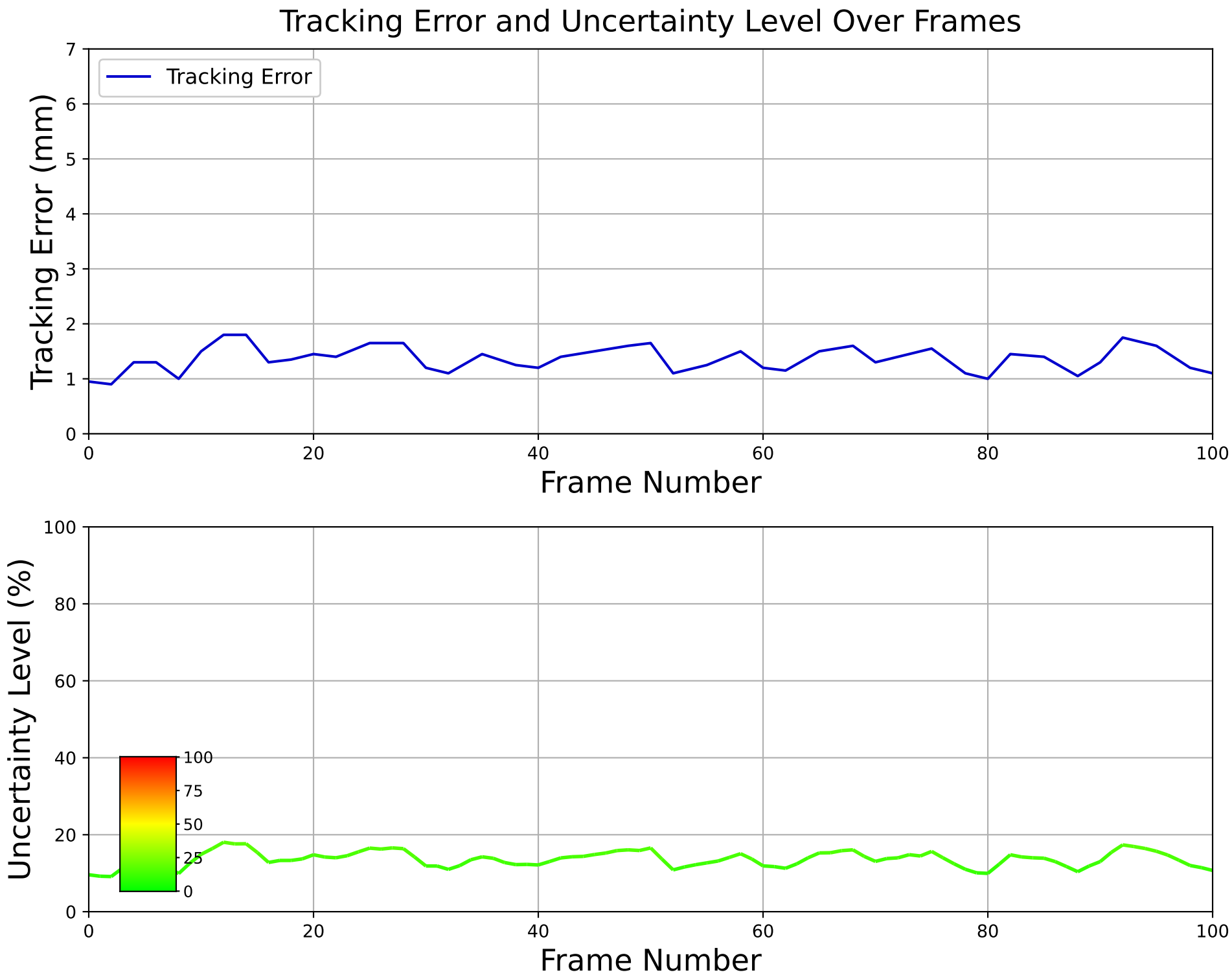

Figure 10: Result plot for a stable condition on clinical dataset (Example 3).

S2.2 Rapid Motion Performance

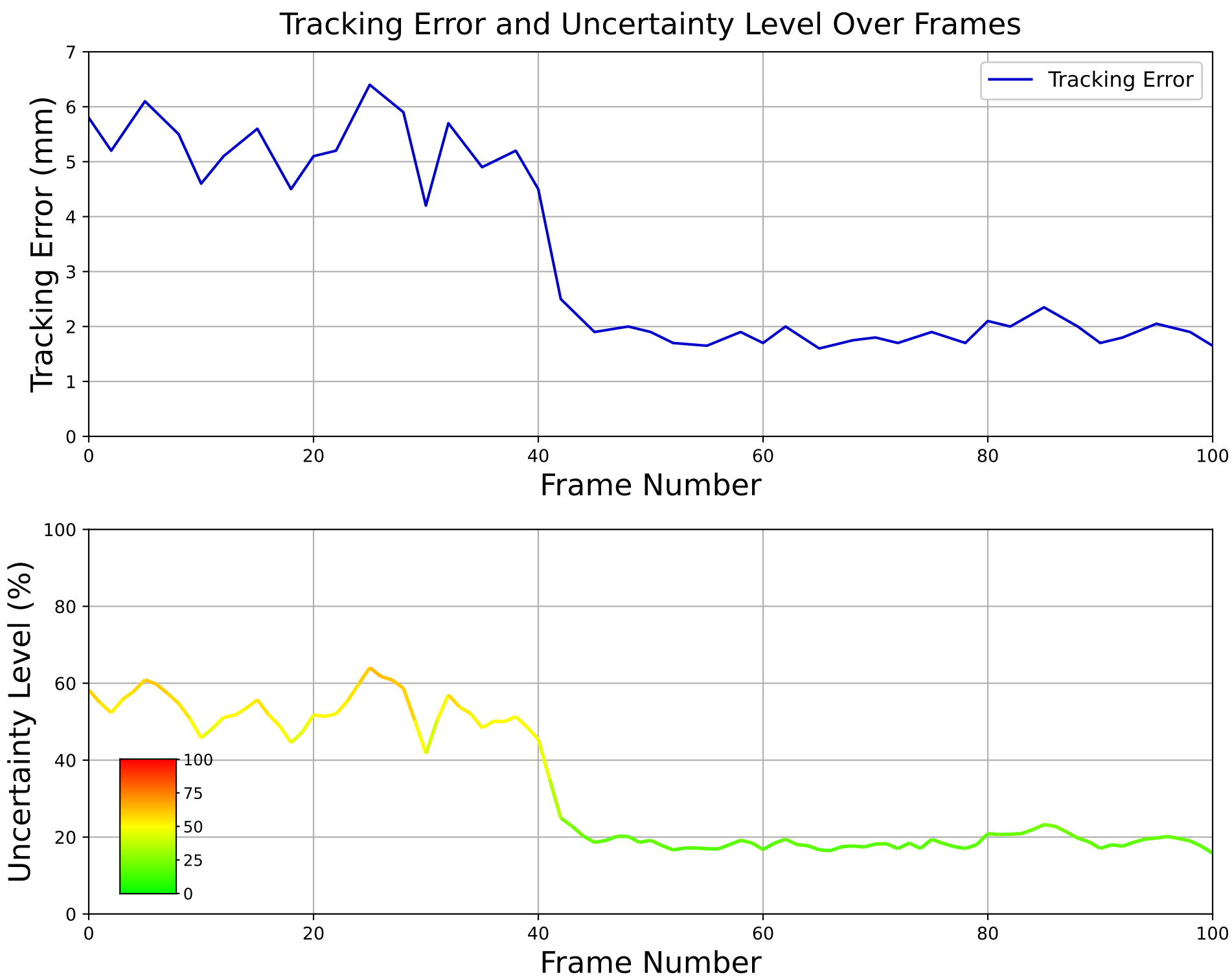

Figure 11: Result plot for a rapid needle movement in the clinical dataset (Example 1).

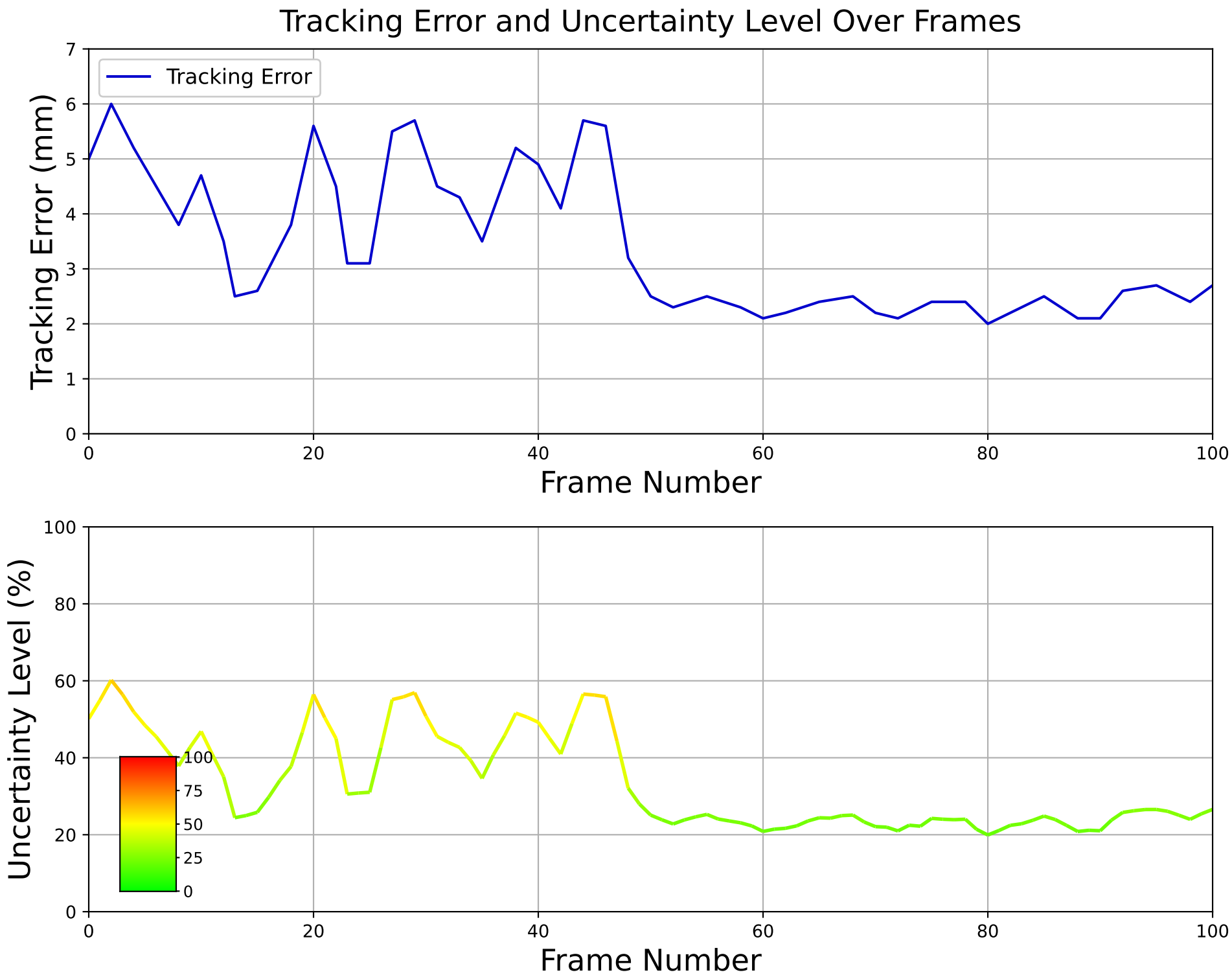

Figure 12: Result plot for a rapid needle movement in the semi-clinical dataset (Example 2).

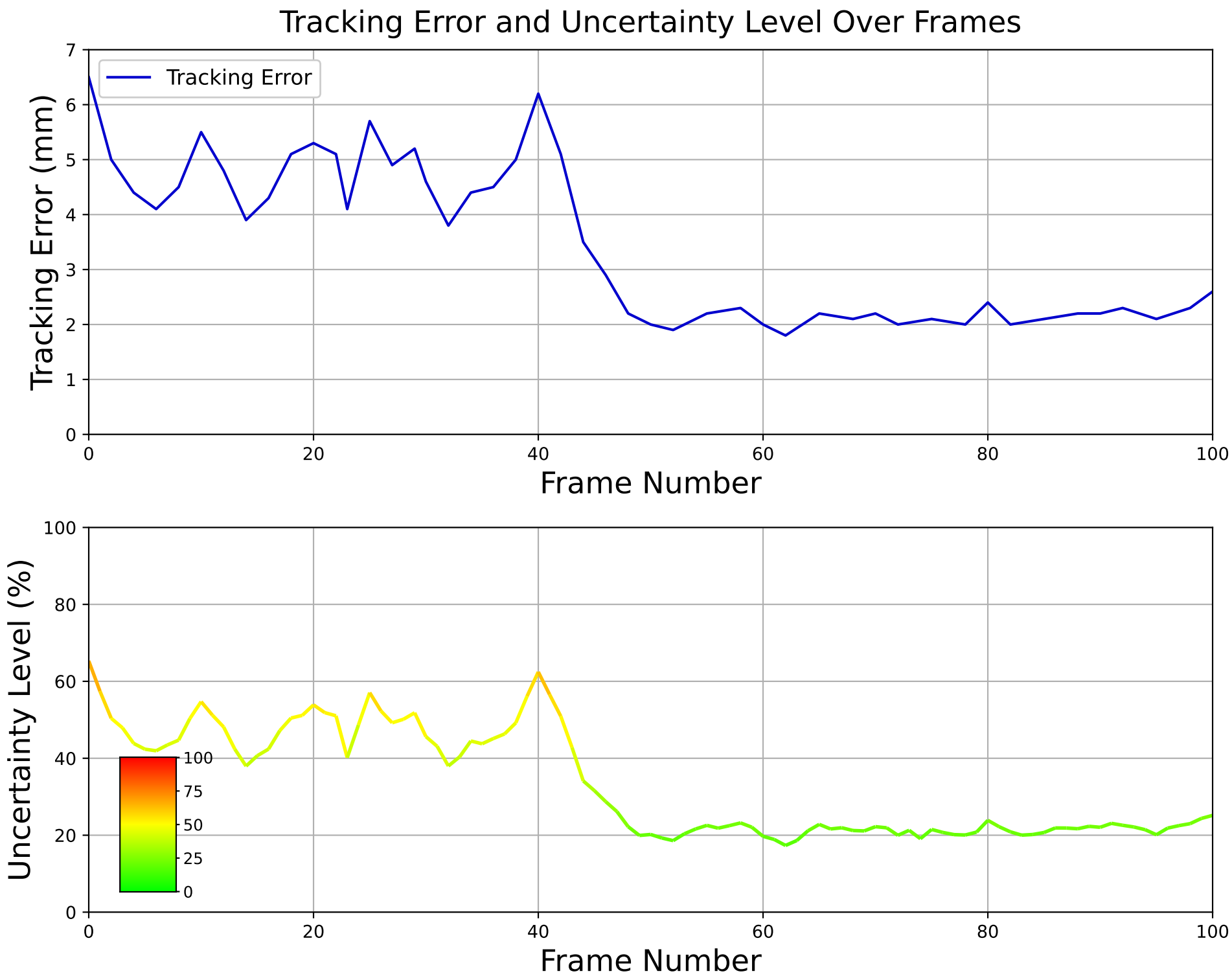

Figure 13: Result plot for a rapid needle movement in the clinical dataset (Example 3).

S3. Detailed Failure Mode Analysis and Limitations

Although the proposed uncertainty assessment framework performs robustly under typical acquisition conditions, there are challenging situations in which the software may produce unreliable uncertainty level estimates or fail to predict accurately. Such limitations can arise, for example, in the presence of severe imaging artifacts, strong needle occlusions, or abrupt out-of-plane motion. The following figures present representative examples of hard cases in which prediction quality can degrade (Figure 14 and Figure 15).

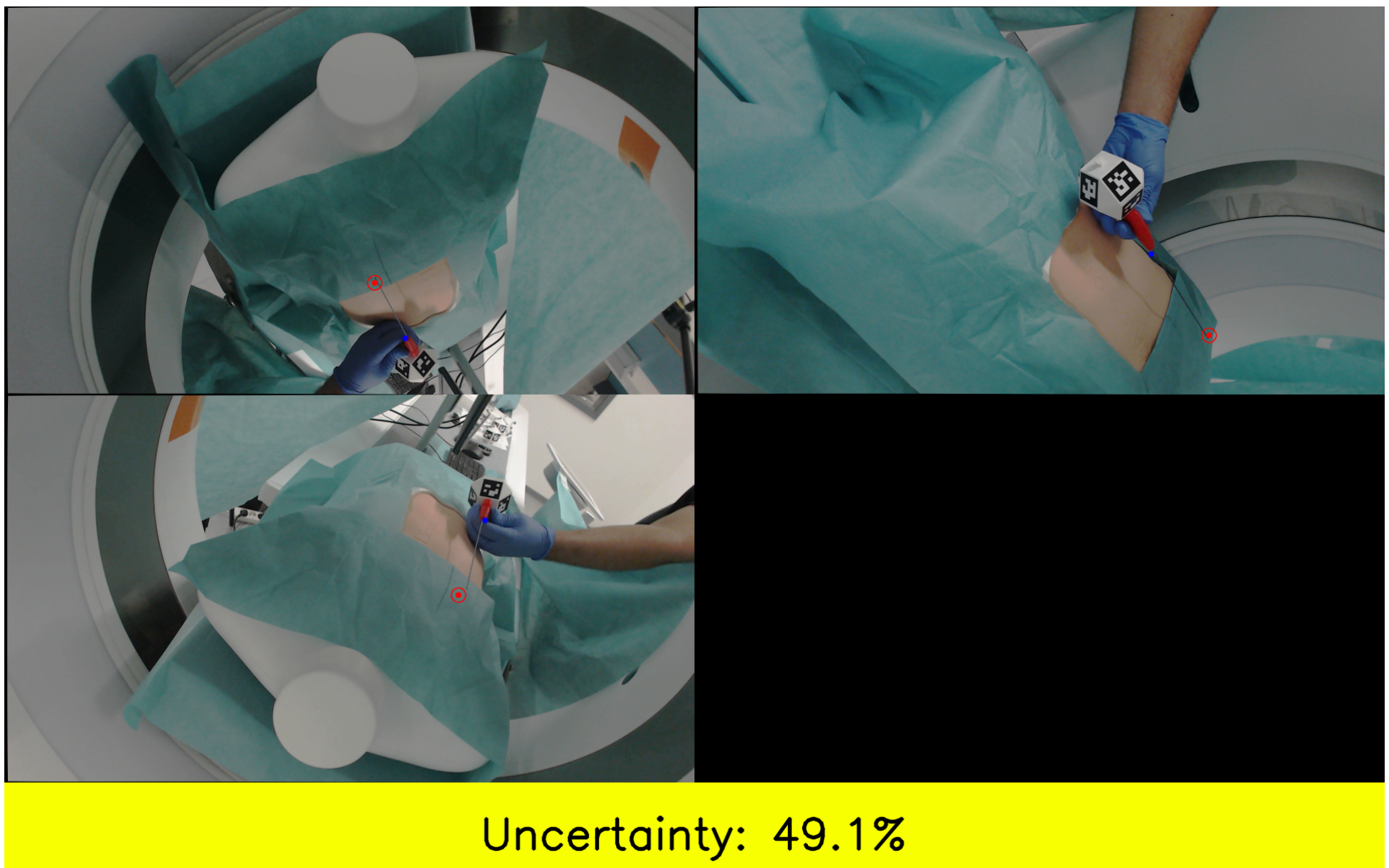

Figure 14: The needle is successfully tracked in at least two camera views; however, the overall uncertainty level remains high due to delayed background updates in the software, leading to inaccurate pose estimation.

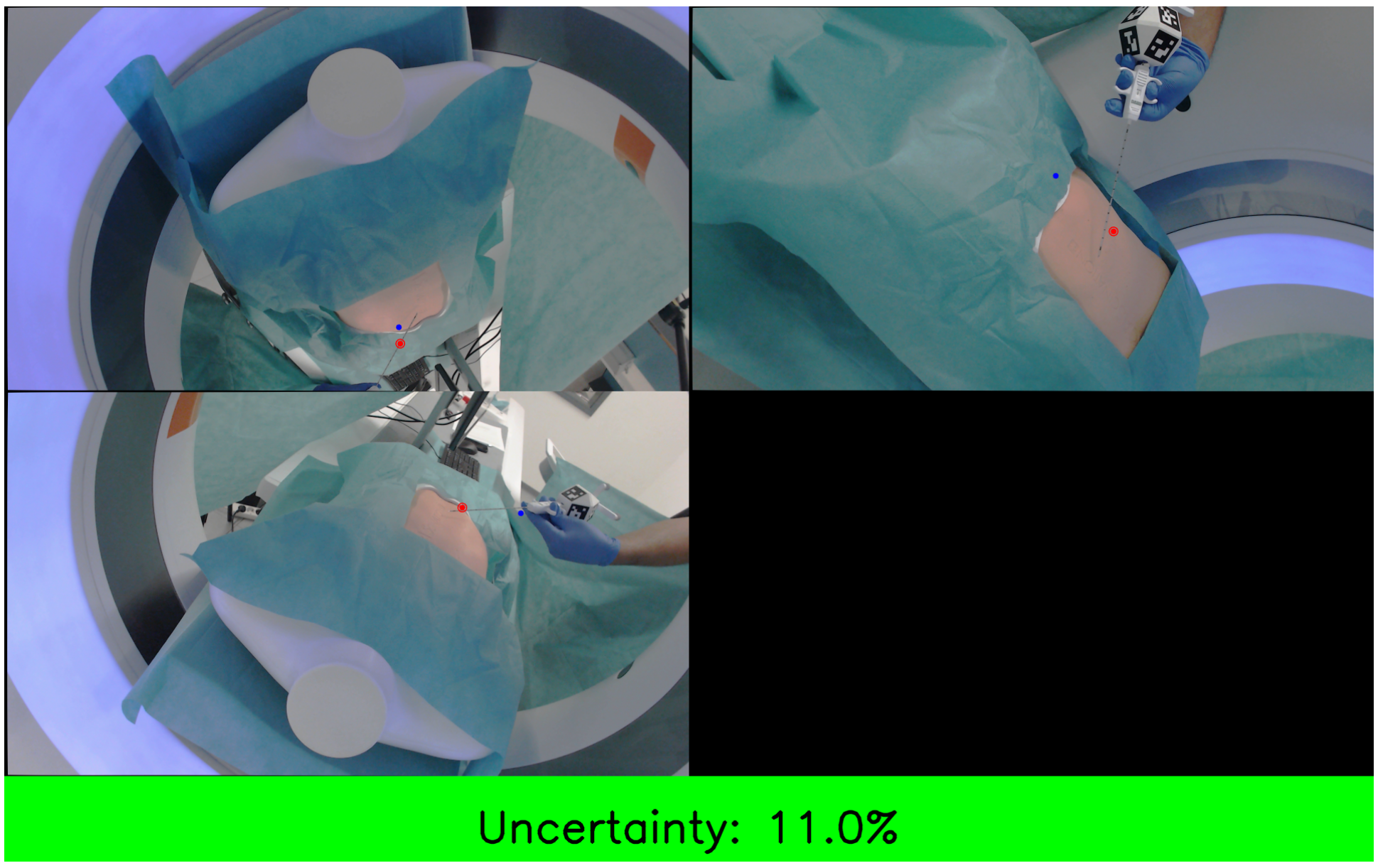

Figure 15: The simultaneous updating of uncertainty level is affected by hardware constraints or unnecessary background computations, leading to occasional delays in the reported values under high system workload.

S4. Supplementary Videos

The video demonstrates the real-time application of the confidence framework in a clinical setting, showing the dynamic changes in uncertainty during movement. The chosen samples present a low-uncertainty case and a dynamic needle insertion and occlusion scenario with tracking failure. In the first scenario, a slow, steady needle movement is shown, in which the tracking error remains low, and no environmental or tracking factors influence the tracking. The uncertainty prediction remains low and stable throughout the movement. In the second scene, needle insertion is shown, altering the needle's appearance and thereby challenging the tracking system. Short spikes of uncertainty are reflected in our system, as evidenced by the tracking. Overall, the system remains low in uncertainty. The last scene depicts a tracking failure and needle occlusion, thereby rendering tracking unusable. While our system responds to the changes and displays high uncertainty, it fails to fully capture the loss of tracking, reaching 100 % uncertainty.
